# Exosomes in diagnostic and therapeutic applications of ovarian cancer

**DOI:** 10.1186/s13048-024-01417-0

**Published:** 2024-05-25

**Authors:** Dhaval Bhavsar, Rajeswari Raguraman, Dongin Kim, Xiaoyu Ren, Anupama Munshi, Kathleen Moore, Vassilios Sikavitsas, Rajagopal Ramesh

**Affiliations:** 1 Department of Pathology, University of Oklahoma Health Sciences Center, 975 NE, 10th Street, Oklahoma City, OK 73104 USA; 2 Department of Pharmaceutical Sciences, University of Oklahoma Health Sciences Center, 1110 N, Stonewall Ave, Oklahoma City, OK 73104 USA; 3 Department of Radiation Oncology, University of Oklahoma Health Sciences Center, 975 NE, 10th Street, Oklahoma City, OK 73104 USA; 4 Department of Obstetrics and Gynecology, University of Oklahoma Health Sciences Center, 800 NE, 10th Street, Oklahoma City, OK 73104 USA; 5 OU Health Stephenson Cancer Center, University of Oklahoma Health Sciences Center, 800 NE, 10th Street, Oklahoma City, OK 73104 USA; 6 Department of Chemical, Biological and Materials Engineering, Oklahoma University, Norman, OK 73019 USA

**Keywords:** Extracellular vesicles, Exosomes, Ovarian cancer, Diagnostics, Drug delivery, Gynecological cancers, Tumor microenvironment, Metastasis, Drug resistance

## Abstract

Ovarian cancer accounts for more deaths than any other female reproductive tract cancer. The major reasons for the high mortality rates include delayed diagnoses and drug resistance. Hence, improved diagnostic and therapeutic options for ovarian cancer are a pressing need. Extracellular vesicles (EVs), that include exosomes provide hope in both diagnostic and therapeutic aspects. They are natural lipid nanovesicles secreted by all cell types and carry molecules that reflect the status of the parent cell. This facilitates their potential use as biomarkers for an early diagnosis. Additionally, EVs can be loaded with exogenous cargo, and have features such as high stability and favorable pharmacokinetic properties. This makes them ideal for tumor-targeted delivery of biological moieties. The International Society of Extracellular Vesicles (ISEV) based on the Minimal Information for Studies on Extracellular Vesicles (MISEV) recommends the usage of the term “small extracellular vesicles (sEVs)” that includes exosomes for particles that are 30–200 nm in size. However, majority of the studies reported in the literature and relevant to this review have used the term “exosomes”. Therefore, this review will use the term “exosomes” interchangeably with sEVs for consistency with the literature and avoid confusion to the readers. This review, initially summarizes the different isolation and detection techniques developed to study ovarian cancer-derived exosomes and the potential use of these exosomes as biomarkers for the early diagnosis of this devastating disease. It addresses the role of exosome contents in the pathogenesis of ovarian cancer, discusses strategies to limit exosome-mediated ovarian cancer progression, and provides options to use exosomes for tumor-targeted therapy in ovarian cancer. Finally, it states future research directions and recommends essential research needed to successfully transition exosomes from the laboratory to the gynecologic-oncology clinic.

## Introduction

Ovarian cancer is the most devastating gynecological cancer and is responsible for more deaths than any other female reproductive cancer [1]. Statistics from the American Cancer Society suggest that a woman has an approximately 1 in 78 lifetime chance of developing ovarian cancer, while her risk of dying from the disease is approximately 1 in 108. Ovarian cancer, based its histological features, is classified into high grade serous carcinoma (HGSC), low grade serous carcinoma (LGSC), endometroid carcinoma (EC), clear cell carcinoma (CCC), and mucinous carcinoma (MC). Among these five histotypes, HGSC is the most aggressive cancer type that is diagnosed in approximately 70% of ovarian cancer patients [2]. Recent advancements in the healthcare field and an early diagnosis have immensely contributed to a better prognosis and improved survival of ovarian cancer patients. However, two-third of patients are diagnosed at an advanced stage of the disease which results in high mortality rates [3, 4], and a 5-year survival rate of only 31% [5, 6]. Currently, cytoreductive surgery followed by chemotherapy using platinum-based drugs is the treatment of choice to manage ovarian cancer [7–9]. The latest National Comprehensive Cancer Network guidelines suggest using intravenous adjuvant therapy with or without intraperitoneal administration [10–12]. However, all the suggested treatments have significant toxicity profiles [13, 14], and drug resistance results in disease relapse [15, 16]. There are numerous ongoing research efforts to improve ovarian cancer treatment, including the potential use of nanomedicine, targeted therapy, immunotherapy, and their various combinations [17]. The progression of these treatment approaches, however, has yet to achieve a cure for ovarian cancer [18]. Thus, there is an urgent need to develop improved methods for the early diagnosis of ovarian cancer and treatment options that limit drug toxicity and combat drug resistance.

Extracellular vesicles (Evs) are lipid bilayer nanovesicles of an endocytic origin secreted by almost all cell types. They mainly include exosomes (30–150 nm, released after fusion of multivesicular bodies with cell membranes), microvesicles (200–1000 nm, assembled and released from the plasma membrane), and apoptotic bodies (1000–5000 nm, released during apoptosis) [19–24]. However, advances made in the field of EVs has led to the identification of additional vesicular particles such as the small ectosomes and arrestin domain-containing protein 1-mediated microvesicles both of which fall in the size range of 30 nm -150 nm but differ in their biogenesis. The lack of specific markers to clearly distinguish exosome from other particles based on their biogenesis has resulted in the usage of the term “exosomes “ for all particles in the range of 30–150 nm. As a result the literature is flooded with studies describing the role of exosomes in cancer pathogenesis and their application in the diagnosis and therapy. For consistency and reproducibility across study reports, the International Society of Extracellular Vesicles (ISEV) upon the recommendation by the Minimal Information for Studies in Extracellular Vesicles, recommended using the terms small EVs (sEVs; < 200 nm) and medium-large EVs (> 200 nm) in lieu of particles that fall within the size range irrespective of their biogenesis. In this article, the reference to exosomes is interchangeable to sEVs owing to the continued usage of the term “exosomes” by numerous researchers, including us [25]. Their composition varies based on the cellular origin; however, exosomes mainly consist of various soluble proteins, antigens, and nucleic acids (such as mRNA and miRNA) contained within an aqueous core surrounded by a cellular membrane and membrane-related proteins. Exosomes play a vital role in cellular signaling via transferring biological molecules from originating cells to receptor cells [26–28]. Early research on exosomes in ovarian cancer patients noted that cancer cells release a higher number of exosomes than normal ovarian epithelial cells [29]. Since then, accumulating evidence from experimental models and clinical datasets supports the role of exosomes in the progression of various cancers, including ovarian cancer. Different researchers indicate that exosomes play a major role in the progression, metastasis, and drug resistance of ovarian cancer [30].

**Fig. 1:**
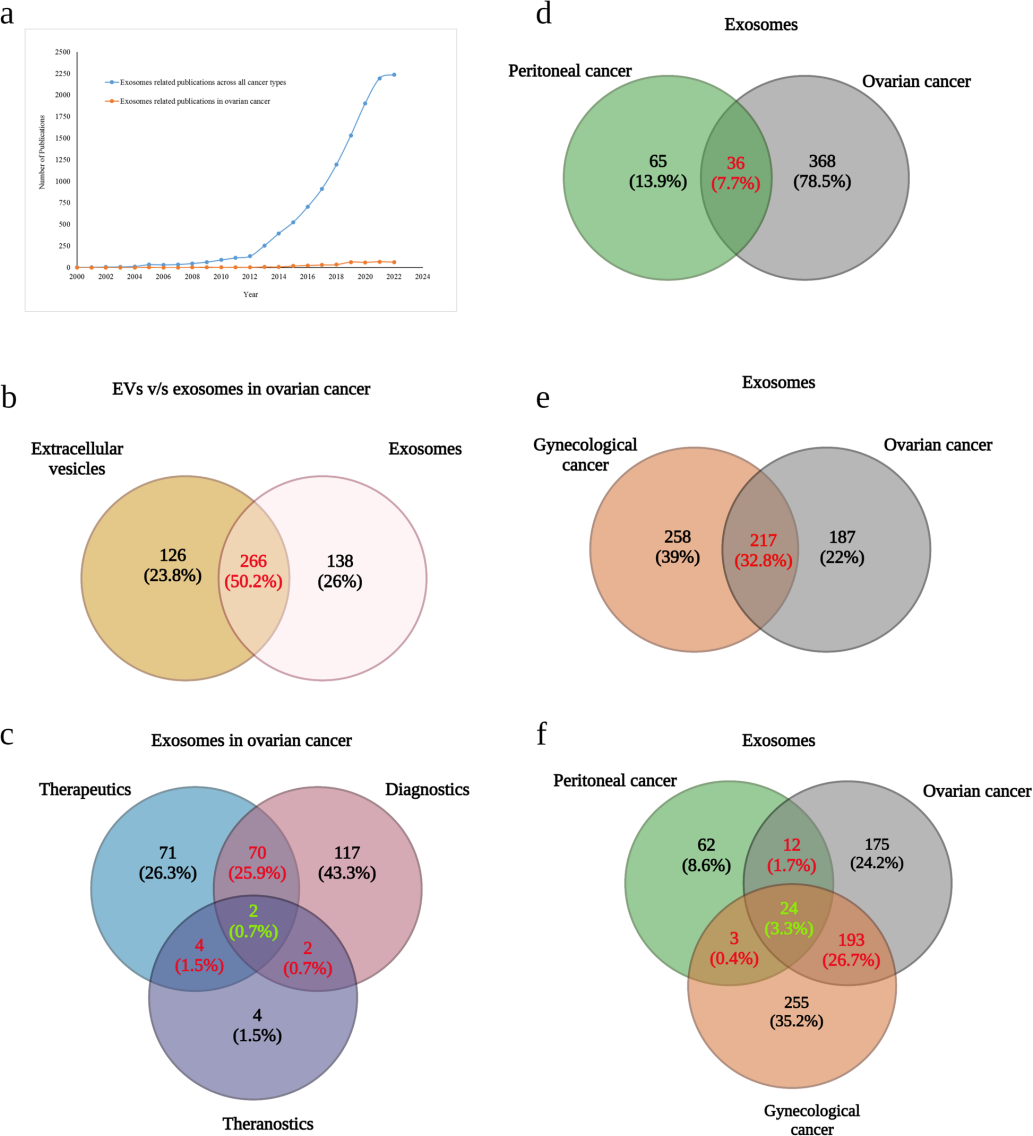
Number of exosome related publications available in PubMed. **a** graph showing number of articles obtained using the search terms “exosomes” and “cancer”, “exosomes” and “ovarian cancer” including review articles categorized year wise. Venn diagram information summarizing number of publications common to input search criteria organized as (**b**) “extracellular vesicles” and “ovarian cancer” v/s “exosomes” and ovarian cancer; (**c**) “exosomes” and “therapeutics” and “ovarian cancer”, “exosomes” and “diagnostics” and “ovarian cancer”, “exosomes” and “theranostics” and “ovarian cancer”. Additionally, the number of exosomes related publications obtained with search terms (**d**) “ovarian cancer” and “peritoneal cancers”; (**e**) “ovarian cancer” and “gynecological cancers” and (**f**) “ovarian cancer” and “peritoneal cancer” and “gynecological cancer” have been compiled. Numbers are reflective of both research and review articles available in the database. Intersection areas are exclusive to publications common under the different input criterion provided. Combining these search criterion gives a comprehensive dataset for a better understanding of number of exosomes related research articles available across multiple cancer types. Figure created with Biorender.com

Exosomes can be found in various body fluids, including blood, urine, saliva, ascites, and cerebrospinal fluid [31]. They are significantly more abundant in cancer patients than in healthy individuals; therefore, exosomal contents potentially reflect the disease pathology and are extensively investigated as candidates for diagnosis and therapy [32, 33]. Furthermore, several recent studies suggest that exosomes possess numerous advantages over conventional drug carriers that make them ideal candidates for next generation drug-delivery systems. There is an increasing trend in the number of exosome-related articles across all cancer types available in PubMed over the last two decades (Fig. 1). Studies investigating exosomes for diagnosis, and prognosis and as a drug carrier in gynecological cancers especially in ovarian cancer is more recent and show an increased trend in the past five-six years (Fig. 1). The relatively new field of exosomal research may potentially overcome the significant barriers to the early diagnosis and efficient treatment of ovarian cancer [30, 34].

As compared to existing available literature, the current review highlights the association between exosomes and ovarian cancer, provides in depth insights about different novel isolation and detection techniques for ovarian cancer-derived exosomes, the emergence of exosomes as a potential biomarker for ovarian cancer, and different possible therapeutic approaches to treat ovarian cancer using exosomes in detail. It also provides insights into the pharmacokinetic properties and the distribution of exosomes in the body, together with their safety and toxicity.

## Ovarian cancer: genetic and epigenetic changes and influence on exosomes

Occurrence of ovarian cancer is multifactorial and is frequently associated with genetic and epigenetic alterations that include oncogene activation, loss of tumor suppressor function, and activation of aberrant cell signaling pathways. For example, mutation in BRCA1 or BRCA2 tumor suppressor genes predispose individuals at risk for developing ovarian cancer and breast cancer [35, 36]. Approximately 14–24% of individuals diagnosed with ovarian cancer harbor BRCA1/BRCA2 mutation and are often hereditary [37]. Additionally, alterations in the proteins (e.g. RAD51C, RAD51D and BRIP1) that complex with BRCA1/BRCA2 contribute to genomic instability, deregulation of cellular machinery and uncontrolled cell proliferation leading to cancer. p53 is another tumor suppressor gene that is frequently mutated in ovarian cancer. About 90% of high-grade serous tumor type harbor p53 mutation with missense mutation being the most common type [38]. Loss of the p53 tumor suppressive function leads to activation of oncogenes and in tumorigenesis and metastasis. Additional gene mutations such as KRas, PTEN and ARID1A have also been reported with some mutations occurring at higher frequency in ovarian cancer sub-types [39–41].

Beyond gene alterations, epigenetic changes that affect gene function and expression also play crucial role in the development and progression of ovarian cancer. Some of the epigenetic changes result in heritable traits [42]. The epigenetic changes reported in ovarian cancer include DNA methylation, histone modification, chromatin remodeling, and non-coding (nc) RNA regulation [43]. A consequence of these changes results in influencing the tumor microenvironment and subsequently the tumor growth, metastasis and response to treatment. DNA methylation occurs in CpG dinucleotide motifs in the DNA sequence and involves the addition of a methyl group to the C5 position of cytosines (5mC). It is the most common and widely investigated epigenetic modification in cancer and includes hypomethylation and hypermethylation. Under hypomethylation state the expression of numerous genes are activated that include increased expression of oncogenes [44]. In contrast, hypermethylation suppresses gene expression including suppression of tumor suppressor genes (e.g. BRCA1, PTEN) that are important for controlling cell cycle and growth [45]. Histones are susceptible to numerous modifications that include acetylation and deactylation, methylation, ubiqutination, and deamination among others. Histone- acetyl transferase (HATs) and deacetyltransferases (HDACs) are enzymes that modulate histone acetylation and deacetylation and are deregulated in human cancers including ovarian cancer [46, 47]. The discovery of ncRNA has led to identification of their importance in the regulation in several cellular processes and their role in cancer. The ncRNA is a large family that includes short interfering (siRNA), long non-coding (lnc) RNA, and micro (mi) RNA among others. The ncRNAs participate in regulating mRNA stability, silencing of mRNA transcription and translation, cell survival, protein function and several other cellular properties. In ovarian cancer, the lncRNAs are over (e.g. HOTAIR, MALAT1)—or under (e.g. TUB4B, GAS5) – expressed and have been assocated with tumor growth, metastasis, angiogenesis, and drug resistance. Changes in miRNA and lncRNA expression influencing ovarian cancer have also been reported [48, 49]. For additional information on ncRNAs and their role in ovarian cancer, readers are advised to refer to the literature.

**Fig. 2:**
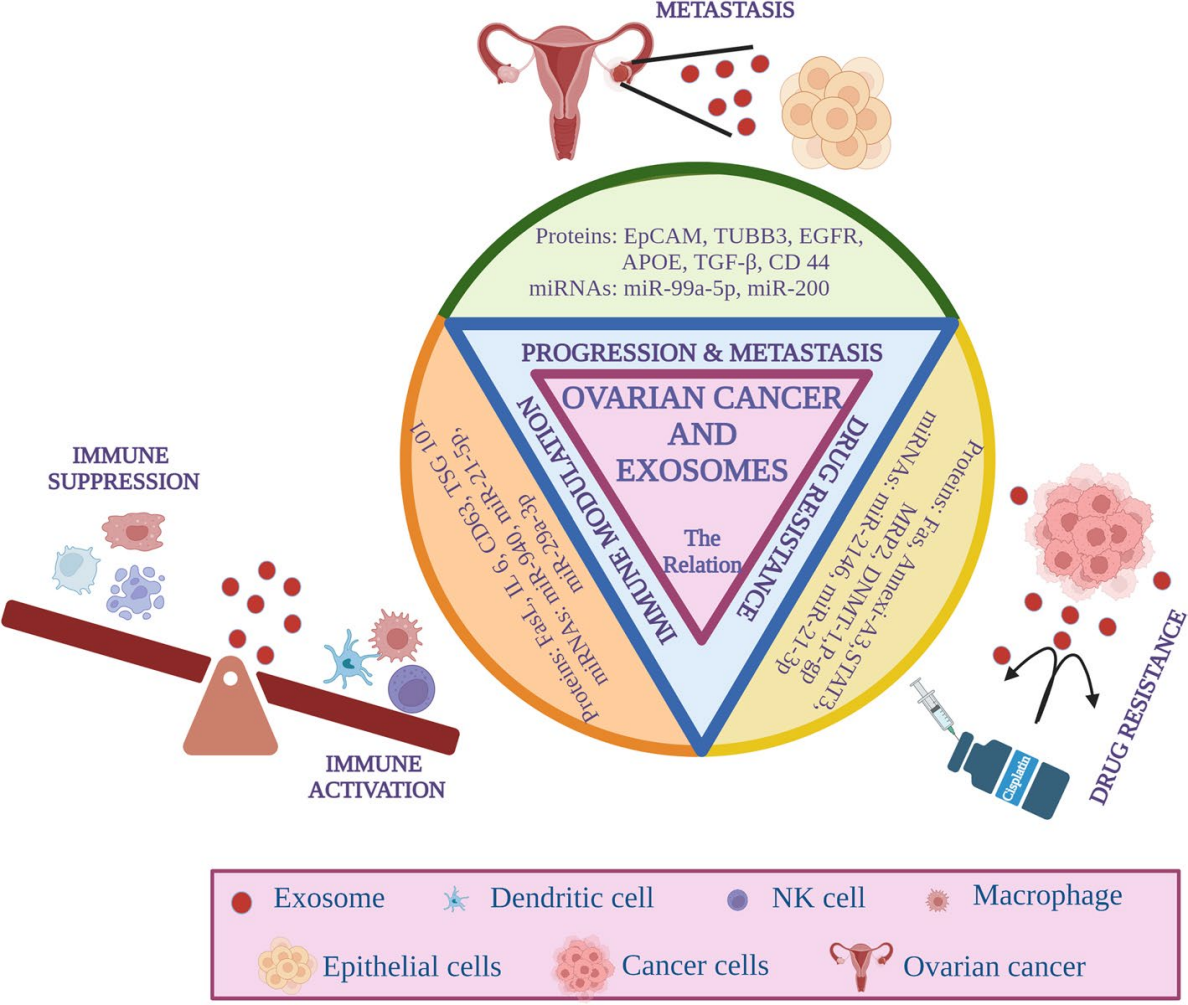
Relationship of exosomes and exosomal contents with ovarian cancer progression and metastasis, immune suppression and drug resistance. Image created with BioRender.com

The molecular and genetic changes brought about in ovarian cancer also influence the packaging of the intracellular contents of EVs especially in exosomes produced the tumor cells. The tumor-derived exosomes (TDEs) in turn play an integral role in promoting tumor growth and spread by communicating with adjacent tumor cells and tumor-associated fibroblasts in an autocine and paracrine manner by transferring their intracellular contents (miRNA, lncRNA, siRNA, protiens, nucleic acid fragments [50]. The exosomal contents can govern various processes of tumor progression such as tumor metastasis (RNAs such as miR-200, miR-99a-5p, and circWHSC1), immune regulation (proteins such as Hsp84/90, MHC I and II, TSG 101, and CD63), and drug resistance (Annexin A3 and miR21; Fig. 2) and discussed in the following sections.

### Exosomes in the progression and metastasis of ovarian cancer

Multiple studies confirm that cancer cell-secreted exosomes can reprogram normal cells to increase tumor survival and foster cancer progression and metastasis [13, 51, 52]. Despite this, research focused on the role of exosomal contents in ovarian cancer progression remains limited [13]. Generally, the proteins found in exosomes secreted by cancer cells significantly differ from those found in normal cell-derived exosomes and can amend the target cell phenotype and boost tumor progression [51, 52]. Proteomics analysis identified various exosomal proteins that significantly contribute to ovarian cancer development and/or tumorigenesis. These include tubulin B-3 chain (TUBB3), epithelial cell surface antigen (EpCAM), proliferation cell nuclear antigen (PCNA), epidermal growth factor receptor (EGFR), claudin 3 (CLDN3), apolipoprotein E (APOE), fatty acid synthase (FASN), LICAM (CD 171), and erythroblastic oncogene B (ERBB2) [53].

Exosomes are an integral part of the tumor microenvironment (TME), which plays an important part in ovarian cancer metastasis [54]. They are predominantly associated with pre-metastatic niche development and promote metastatic progression. Exosomes derived from primary ovarian cancer cells can reconfigure the TME through intercellular transmission between cancer cells and normal stromal cells, immune cells, and cancer-associated fibroblasts (CAFs) to stimulate metastatic incursion [55]. These exosomes also transform fibroblasts (a key player in homeostasis and normal tissue functioning) into CAFs [56]. Additionally, ovarian CAF-derived exosomes carry transforming growth factor β-1 (TGF-β1). This augments the potential of ovarian cancer cells to migrate and invade and promotes epithelial-mesenchymal transition (EMT) by SMAD signaling activation [57]. Mesenchymal stem cells derived from adipose tissue (ADSCs) when treated with exosomes derived from two ovarian cancer cells (OVCAR-3 and SK-OV-3) show distinctive features of tumor-linked myofibroblasts as characterized by elevated expression of α-smooth muscle actin, TGF-β, stromal derived factor-1 (SDF-1), and TGF-β receptor I and II. Additionally, there is a difference in the signaling pathway modulated under each setting. SK-OV-3-derived exosomes function via the SMAD-dependent pathway, while OVCAR-3-derived exosomes increase phosphorylated AKT levels via a SMAD-independent pathway. Together, these data underscore the significant contribution of exosomes derived from ovarian cancer in the formation of tumor-associated myofibroblasts to create a suitable microenvironment for the progression and invasion of cancer cells [58]. In addition, various studies support the role of exosomes in promoting the invasive abilities of cells. Morphological changes and increased invasion capacity of human peritoneal mesothelial cells (HPMCs) are observed upon treatment with CD44 expressing tumor cell-derived exosomes. Additionally, transfer of CD44 from the cancer cell-derived exosomes to HPMC induced the secretion of matrix metalloproteinase-9 (MMP-9) that dispersed the mesothelial barrier and promoted cancer invasion [59]. In contrast, HPMCs treated with exosomes derived from normal immortalized ovarian surface epithelial (IOSE) cells did not show any effect. In another study, higher expression of CD24, EpCAM, and proteolytic enzymes (pre-MMP2 and pre-MMP9) in malignant ascites-derived exosomes was reported [60]. The presence of MMPs in the exosomes would help degrade the ECM and facilitate tumor invasion.

Likewise, exosomes from cancer cells transfer miRNA to the stromal cells present in the TME which contributes to tumor progression. Increased levels of exosomal miR-99a-5p derived from epithelial ovarian cancer cells (TYK-nu and HeyA8) causes HPMCs to increase the expression of fibronectin and vitronectin, thereby promoting tumor invasion [61]. Claudin 4 is overexpressed in exosomes derived from BG-1 ovarian cancer cell line and in serum from ovarian cancer patients; this protein controls the permeability of the paracellular barrier and increases metastasis potential and its suppression reduces invasion and metastasis [62–64].

In summary, cell–cell communication mediated by tumor cell-derived exosomes and transfer of their cargo significantly contribute to ovarian cancer invasion and metastasis.

### Exosomes in immune modulation

**Fig. 3:**
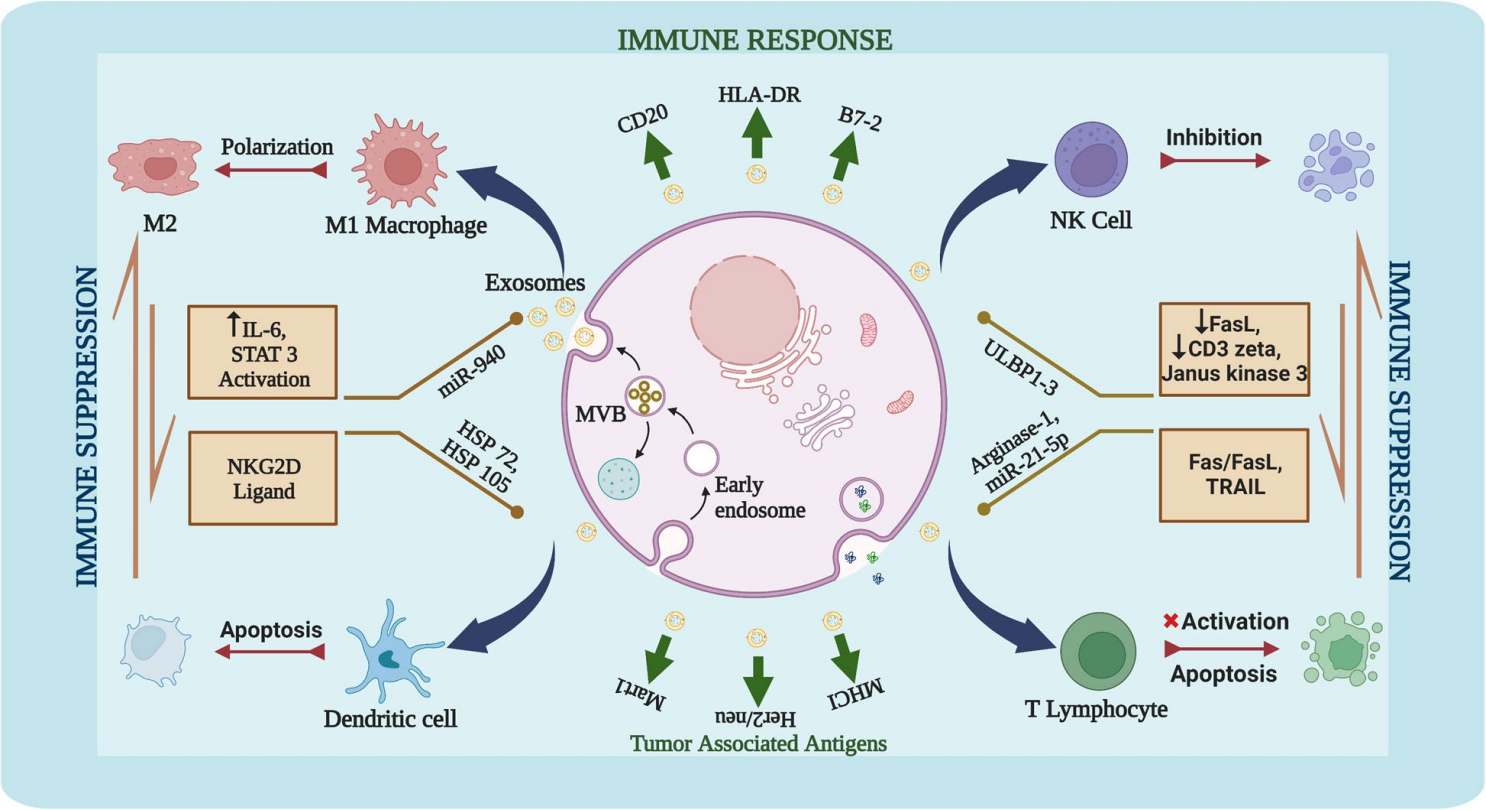
Role of exosomes in immune modulation in ovarian cancer. The antigen carried on the surface of the exosomes may activate an immune response, while different exosomal contents may suppress the immune system or help to evade immune recognition. Image created with BioRender.com

A variety of immune cells [including natural killer cells (NK cells), macrophages, and T and B lymphocytes] operate ubiquitously in the TME and various proteins and RNAs regulate their function. Exosomes are a universal component of the TME and play a critical role in the crosstalk among tumor cells and the immune system. Specifically, cancer cell-derived exosomes support cancer progression by helping cells escape the immune system in several ways (Fig. 3). Hence, their potential role in immune modulation has attracted researchers’ attention [65, 66]. Cancer-associated exosomes exert immunosuppressive action via several mechanisms, including T lymphocyte apoptosis via the Fas/Fas ligand pathway and reducing NK cell activity through NKG2D ligands [67–69]. Exposure of peripheral blood mononuclear cells (PBMC) and dendritic cells (DCs) to ovarian cancer cell-derived exosomes induced apoptosis via the Fas/FasL pathway and TNF-related apoptosis-inducing ligand (TRAIL) [70]. Additionally, exosomes isolated by differential centrifugation from ovarian cancer patients' amniotic fluid or ascites activate Toll-like receptor to boost interleukin-6 (IL-6) production in monocytes and activate the STAT3 pathway in tumor cells, stromal cells, and immune cells. Together, this helps tumor cells escape the immune system [71]. Exosomes derived from the ascites of ovarian cancer patients by applying chromatography/centrifugation or density gradient centrifugation method, induces T-cell apoptosis in vitro by suppressing key components in the T cell activation pathway: Janus kinase 3 and CD3-zeta [72]. Similarly, Arginase-1-containing exosomes isolated using sequential centrifugation from ascites and plasma of ovarian cancer patients contributed to tumor progression and immune escape by suppressing the CD3-zeta and -epsilon chain in T-cells and reduced proliferation of CD4 + and CD8 + T-cells in vitro and in vivo [73]. Exosomes isolated by size exclusion chromatography and ultracentrifugation from the plasma of ovarian cancer patients potentially mediate the conversion of CD4^+^CD25^neg^ T cells into CD4^+^CD25^high^ FOXP3^+^ Tregs, which inhibits the function of CD8^+^ T cells and CD4^+^CD25^neg^ T cells. These exosomes encourage Treg proliferation and increase immunosuppressive function through mechanisms associated with TGF-β and IL 10 [74]. Reconfiguration of immune system also occurs by transfer of miRNAs from cancer cell-derived exosomes to tumor associated macrophages (TAMs). miR-222-3p from ovarian cancer cell (SKOV3)-derived exosomes when transferred to tumor associated macrophages (TAMs) induced polarization to the immunosuppressive M2 phenotype and involved the SOCS3/STAT3 signaling pathway [75]. miR-940 levels under hypoxia is increased in cancer-derived exosomes and uptake of these exosomes by unpolarized TAMs undergo transformation to the M2-like phenotype [76, 77]. Exosomes-derived from TAMs also contribute in promoting the immunosuppresive environment by transferring STAT3-targeting miRNAs (miR-21-5p and has-miR-29a-3p) to T cells and increasing the Treg/Th17 ratio and tumor progression. Conversely, silencing of the two miRNAs with miRNA mimics inhibited STAT3 and reversed the Treg/Th17 ratio to a immune favorable environment and inhibition of tumor growth [78]. All of these study results demonstrate exosomes foster tumor progression by modulating the immune response.

While the role of exosomes in tumor growth and metastasis has been extensively studied, very few studies have described the role of exosomes in stimulating the immune system in ovarian cancer. Considering the immune-promoting action of exosomes in other diverse tumor, ovarian cancer-derived exosomes may also modulate the innate and adaptive immune systems through DC, NK-, or cytotoxic T-cell activation [79]. Generally, exosomes containing tumor-associated antigens (TAAs) are identified by antigen-presenting cells such as dendritic cells and macrophages and presented to T cells for stimulating the anti-tumor activity of immune cells [34, 80, 81]. Priming of dendritic cells with TAA (MHC I, HSP70, HSP90, Mart1, and Her2/Neu) carrying exosomes derived from ovarian cancer ascites and subsequent exposure to resting T cells resulted in T cell activation and increased tumor cell cytotoxicity [82].

In conclusion, the exact role of exosomes in modulating the immune system in ovarian cancer remains unknown, and more research is required to confirm their role as stimulators and/or suppressors of the immune system.

### Exosomes promote chemotherapeutic resistance

Drug resistance is considered a major reason for the failure of cancer therapy. Therefore, it is necessary to explicate the molecular mechanism(s) of cancer progression and therapeutic resistance to design novel, effective therapeutic schemes. Metastasis or resistance to chemotherapy is the key reason for the observed therapeutic failure in ovarian cancer patients, specifically at advanced stages. Recently, exosomes were identified as key players in the development of drug resistance in several malignancies, including ovarian cancer [83–86]. There is an inverse relationship between intracellular drug concentration and the level of secreted exosomes in the context of ovarian cancer; this confirms the crucial role of exosomes in chemoresistance [79]. Ovarian cancer patients that respond to chemotherapy have altered exosomal protein levels, while exosomal protein levels are unchanged in unresponsive patients [87]. Thus, exosomal protein contents may be a valuable tool to predict the therapeutic response. The three recognized mechanisms wherein exosomes promote drug resistance in ovarian cancer are briefly described in the following sections.

#### Transfer of miRNAs to modify gene expression and exert resistance

Exosomes modulate chemoresistance in cancer cells by multiple mechanisms that includes transfer of miRNAs. The role of exosomal miRNAs in chemoresistance was delineated using paclitaxel and cisplatin in experimental models. For example, miR-1246 targets the Cav-1/p-gp/M2-type macrophage axis leading to paclitaxel resistance in ovarian cancer cells. Meanwhile, combination treatment using chemotherapy and an miR-1246 inhibitor significantly reduced the tumor burden in vivo [88]. The transfer of miR-21 from cancer-associated adipocytes and cancer-associated fibroblasts (CAFs) to cancer cells through exosomes suppresses apoptosis and promotes resistance to paclitaxel in ovarian cancer cells by targeting apoptotic protease activation factor 1 [89]. Exosomal miR-21-3p suppresses neuron navigator (NAV) 3 levels which may increase the resistance of ovarian cancer cells to cisplatin [90].

#### Transfer of proteins that export drugs out of cells

Studies on the connection of exosomal proteins and chemoresistance in ovarian cancer reveal that exosome-mediated transfer of proteins promotes resistance by inhibiting apoptosis or enhancing drug efflux [79, 91–96]. Annexin A3, P-glycoprotein (P-gp), signal transducer and activator of transcription 3 (STAT3), FAS, DNA methyltransferase-I (DNMT-1), multidrug resistance protein 2 (MRP2), ATP 7A, and ATP 7B are exosomal proteins that promote drug resistance in ovarian cancer cells [79, 91–96]. Hypoxia-derived exosomes contain high amounts of STAT3 and FAS proteins that induce drug resistance. Increased levels of Annexin A3 in platinum-resistant ovarian cancer cell lines impart drug resistance by reducing the concentration of platinum compounds within the cell and inhibiting apoptosis. The confirmed presence of Annexin A3 in exosomes indicates that they are involved in the intercellular transfer of Annexin A3 to impart resistance [91, 92]. P-gp promotes drug efflux, and the expression of P-gp is higher in exosomes derived from platinum-resistant A2780 cells than in those derived from platinum-sensitive wild-type A2780 cells [93]. Elevated levels of DNMT1 in exosomes and their contribution to drug resistance has also been reported [96]. Exosomes from ovarian cancer patients and SKOV3 ovarian cancer cell line highly enriched with DNMT-1 protein levels abrogated the cytotoxic activity of cisplatin and enhanced tumor growth in vivo. However, depletion of DNMT-1 enriched exosomes with GW4869, a nSNMase2 inhibitor, restored cisplatin sensitivity both in vitro and in vivo [96]. The results from these studies demonstrate a role for exosomal proteins in contributing to resistance to anticancer drugs.

#### Induction of EMT characteristics

EMT is known to promote tumor cell migration and invasion and contribute to establishment of metastasis at distant site. Studies exist demonstrating EMT as one of the contributory mechanisms for drug resistance and reversing the EMT phenotype reverts sensitivity to anticancer drugs [97–99]. Epimorphin also known as syntaxin-2 belongs to the SNARE family of proteins and plays an important role in the cell morphogenesis and protein transport [100, 101]. To determine if reversal of MET phenotype with epimorphin will restore sensitivity to cisplatin, a panel of ovarian cancer cell lines (A1847m, A2780, OVCAR10) were treated with epimorphine and observed for drug sensitivity. Epimorphine treatment increased tumor cell sensitivity to platinum drugs owing to the reversal of mesenchymal to epithelial phenotype [102]. In another study, treatment of A2780 ovarian cancer cells with exosomes derived from three platinum-resistant derivatives of A2780 (C30, CP70, and C200) exhibited a considerable decrease in the expression of epithelial markers (E-cadherin, EpCAM, and dystroglycan) and an increase in the expression of mesenchymal markers (TWIST, vimentin, and paladin), and indicator of EMT phenotype that coincided with resistance to carboplatin [103]. These findings confirm the role of exosomes in the induction of EMT and consequently in drug resistance in ovarian cancer. Further studies are required to elucidate other likely mechanisms of exosome-mediated drug resistance.

### Exosomes promote stem cell modification

Cancer stem cells (CSCs) can escape chemotherapy and undergo self-renewal, and differentiation leading to tumor recurrence and failure to therapy [104]. Ovarian cancer is highly aggressive, often recurrent and drug-resistant in nature, and CSCs is a contributing factor to this recurrent and drug-resistant behavior [105]. Recent evidence shows that ovarian cancer cells (SKOV3 and CoC1) have elevated viability and stemness following treatment with exosomes derived from MDA-MB-231 breast cancer cells. Molecular studies demonstrated treatment of SKOV cells with MDA-MB-231-derived exosomes increased miR-454 expression in CD44 + /CD133 + SKOV cells through the activation of the Wnt pathway via proline rich transmembrane protein 2 binding and increased stemness in vitro and tumor growth in vivo [106]. Use of C59, a Wnt inhibitor reversed stemness and increased tumor cell cytotoxicity. The results showed the ability of tumor-derived exosomes to modulate stemness and promote tumor growth. Exposure of ovarian cancer stem-like cells to ascites-derived exosomes showed maintenance of stemness and promote invasive propertise of the tumor cells [107]. While reports on the role of cancer cell-derived exosomes on CSCs modification and promotion are emerging, they remain very limited in ovarian cancer and warrants further investigation.

## Exosome isolation approaches

**Fig. 4:**
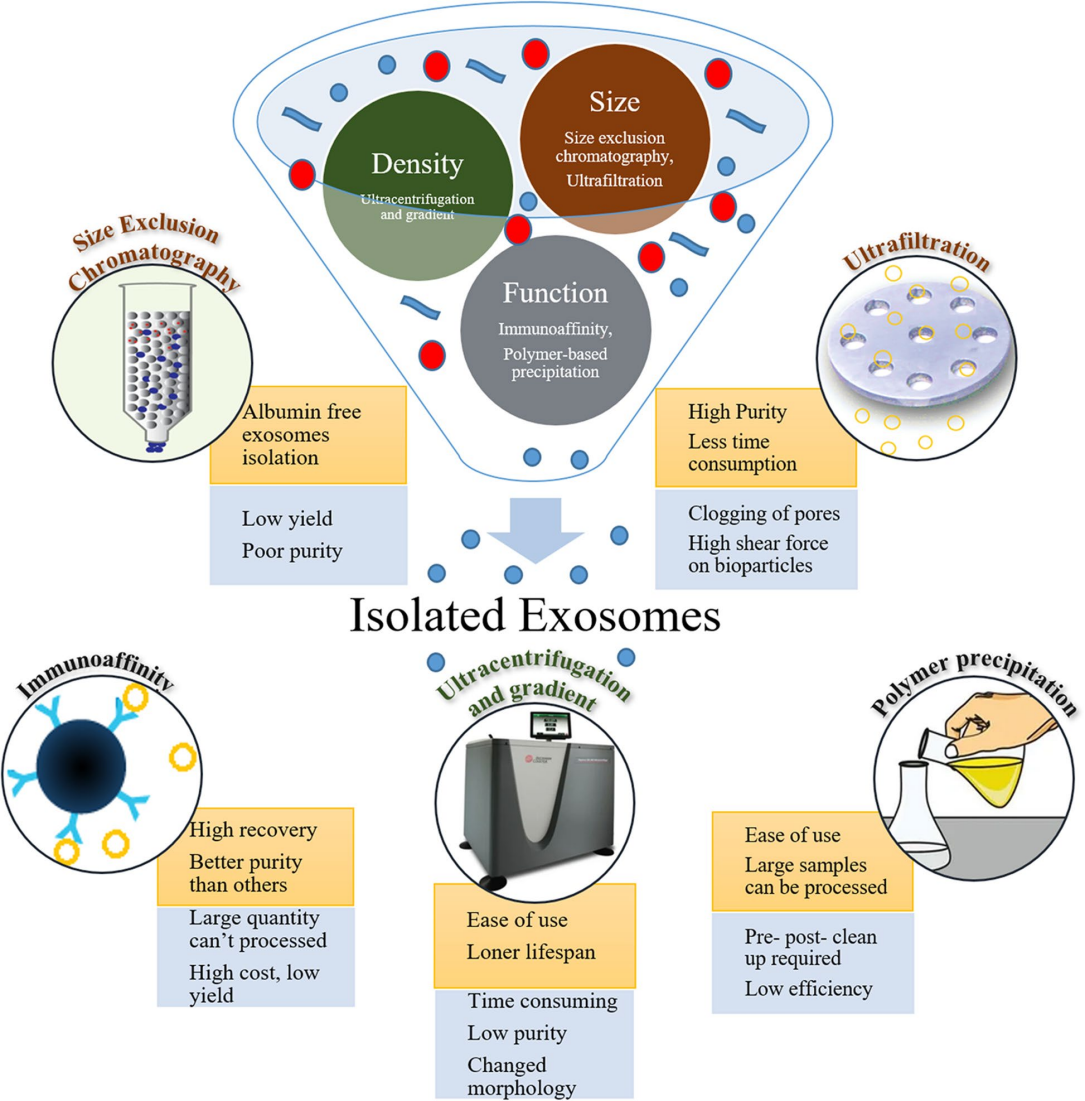
Overview of conventional exosome isolation methods with its merits and demerits. Image created with BioRender.com

Exosome isolation and purification are prerequisites for diagnostic and therapeutic applications. However, the technology to consistently isolate highly purified exosomes to homogeneity is far from standardized [108]. The presence of other vesicles including exomeres, large EVs and microvesicles among others make it difficult to isolate pure exosomes from biological samples [109, 110]. Conventional methods to isolate and purify exosomes such as ultracentrifugation, ultrafiltration, size exclusion chromatography (SEC), polymeric precipitation, and immunoaffinity are well established and widely used. However, they are time consuming, require bulk amounts of samples, and produce low-purity materials [111]. Therefore, there is a need to develop novel exosome isolation methods. Summarized below are the techniques used in studying exosomes on ovarian cancer. Readers are informed that the description of the purification processes described below is limited to investigating exosomes in ovarian cancer and is not comprehensive as several other techniques exist and are directed to refer to the literature. Figure 4 summarizes various conventional exosome isolation methods with their merits and demerits.

Ultracentrifugation is the most widely utilized approach to separate exosomes from impurities. This technique separates exosomes from other particles based on their sedimentation rate due to their different densities, sizes, and shapes [112, 113]. This process offers advantages such as ease of use, an established protocol, the ability to process most samples and a low cost [112, 113]. However, this entire process is time consuming [81, 114, 115], and low yielding with poor reproducibility [116], and contains impurities that may affect further follow up analyses, even after multiple centrifugation steps [116, 117]. Moreover, the prolonged high-speed centrifugation may damage the exosomes and alter their morphological and biological functions [116, 117]. Size exclusion chromatography (SEC) (otherwise known as gel filtration) utilizes a column with porous beads of a specific pore size and separates the samples based on their sizes [118]. The larger sized particles cannot pass through the pores and elute faster than smaller particles that enter the pores and elute at a later time. Thus, selective elution and separation of particles based on size are achieved [108]. Exosomes from ovarian cancer patients that were isolated using SEC have an almost identical size, shape, and protein composition with those prepared using ultracentrifugation [72].

Exosome purification can also be performed using immunoaffinity and polymer-based precipitation approaches depending on their functional characteristics [119]. Immunoaffinity approaches utilize the interaction of a specific antibody and antigen to separate and purify exosomes. All exosomes have specific proteins on their surfaces (such as tetraspanins and annexins) that can interact with explicit antibodies, polysaccharides, or peptides to facilitate their “pull down” and separation from other biological components [113, 120–122]. A significantly high amount of EpCAM-positive exosomes were isolated from the sera of patients with benign diseases compared with that from the sera of healthy individuals using EpCAM modified magnetic activated cell sorting process [123]. Furthermore, the amount of EpCAM-positive exosomes increases as the disease progresses. The immunoaffinity approach is highly specific and capable of precisely isolating subclasses of exosomes. However, it is time-consuming, costly, and unable to process large quantities of sample and may result in low purity owing to potential isolation of other biological vesicles with similar proteins [113, 121].

The polymer precipitation method is widely utilized in commercial kits. The exosome solubility/dispersibility is altered with the help of different hydrophilic polymers, and polyethylene glycol is the most widely used [124, 125]. The exosomes are precipitated and collected after low-speed centrifugation following the addition of these polymers. This method (ExoQuick precipitation) was used to isolate sEVs from SKOV3 cells. The purified exosomes are spherical and 50–150 nm in size, and are enriched with exosome-specific markers: CD9, CD63, and CD81 [126]. This approach is simple and fast and does not require any specialized instruments, other approaches. However, the main shortcoming is precipitaion of other proteins, lipoproteins, and polymeric material that can reduce the efficiency of exosome isolation [112]. All the conventional techniques used to isolate exosomes have merits and demerits; it is important to compare these methods to determine the best conventional method to efficiently isolate high-purity ovarian cancer-derived exosomes. The ExoQuick precipitation method generates the highest quantity and purity of exosomal RNAs and proteins from the exosomes of ascites derived from ovarian cancer patients compared with other techniques. Ultracentrifugation yields the least pure exosomes with the lowest content of RNAs and proteins. The purity of exosomal RNA is similar in exosomes isolated using SEC and EpCAM dynabeads (an immunoaffinity approach), although there is a much high number of isolated exosomes using SEC. Meanwhile, similar amount of proteins is isolated using EpCAM dynabeads, SEC, and ultracentrifugation [127].

Conventional approaches are most widely used for exosome isolation; however, they have many limitations that restrict their ability to meet the growing scientific needs. Membrane-based and microfluidics-based techniques are emerging approaches to isolate exosomes [111]. Exosomes are lipid bilayer nanovesicles; therefore, their surface is enriched with the negatively charged lipid, phosphatidylserine. This presents a possibility for innovative techniques [128]. The expression of phosphatidylserine is the highest in exosomes derived from the plasma of ovarian cancer patients with malignant tumors, followed by those of patients with the benign disease and healthy individuals [129]. Thus, the isolation approaches using phosphatidylserine might benefit ovarian cancer patients.

Innovative microfluidics-based approaches were recently developed to purify exosomes [121]. Microfluidics utilizes a small lab-on-a-chip platform consisting of micron-sized channels, to process micro to picolitre amounts of samples [111, 130]. Microfluidics technology rapidly isolates exosomes at a high level of sensitivity and purity with a reduced cost using few reagents in a minimal time. Different techniques have been developed for size-based isolation of exosomes using microfluidics [131–134]. Electrical-based isolation techniques depend on the intensity of the electric field, size, and the electric properties. An AC electrokinetic-based platform was used to isolate exosomes from the plasma of early stage (stages I and II) ovarian-, pancreatic-, and bladder cancer patients in accordance with the ISEV 2018 guidelines [135]. A conventional immunoaffinity approach is usually incorporated in the microfluidic device to isolate exosomes based on their function. Immunoaffinity approaches depend on the interaction between antigens and antibodies; therefore, the key factors to efficiently isolate exosomes include appropriate antibody selection, microscale bulk transfer stimulation, and optimized collisions between the particle and surface [136, 137]. This is mostly achieved using two methods: 1) *Microchannel surface modification with appropriate antibodies*. This type of microfluidic method was used to isolate exosomes from the sera of high-grade serous ovarian cancer patients [138]. The inner surface of the microchannel was modified by covalent attachment of antibodies (anti-EpCAM or anti-CD9) against cancer and normal exosome membrane biomarkers. This is an inexpensive, rapid, and specific method to obtain intact and label-free exosomes. It results in a higher yield from a minimal amount of sample than does other conventional isolation method (ultracentrifugation and Exoquick followed by conventional immunoaffinity). Furthermore, the increase in the amount of EpCAM + exosomes positively correlates with the progression of high-grade serous ovarian cancer [138]. Similarly, functionalization of the inner microchannel surface with anti-CD63 and anti-EpCAM antibodies to isolate exosomes from conditioned culture medium (OSE, FTSEC, and OVCAR-8 cell lines) results in a significant increase in the amount of exosomes obtained from OVCAR-8 cells [139]. This technique is the quickest, the most cost effective, and specific and results in the highest yield compared with conventional approaches. 2) *Addition of magnetic beads or other affinity particles*. An Exosearch chip was designed for the quantitative isolation of exosomes from the plasma of ovarian cancer patients using antibody-conjugated magnetic beads in a microfluidics platform [140]. The exosomes isolated with Exosearch have a narrow size distribution range, and the technique is more specific than ultracentrifugation. The application of this system for blood-based diagnosis of ovarian cancer results in highly specific and simultaneous detection of three tumor antigens within the same exosome subpopulation. It accurately distinguishes between exosomes from the plasma of ovarian cancer patients and that of healthy individuals. The developed chip is a low-cost and convenient approach for the specific, rapid isolation of blood diagnostic exosomes.

Thus, the emerging lab-on-chip platforms are promising approaches capable of merging multiple necessary steps such as sample loading, processing, and detection for the downstream analysis of specific RNAs and proteins on a single device that facilitates the clinical translation of extracellular vesicle analysis.

## Novel technologies for exosome analysis in ovarian cancer

Exosomes are routinely characterized for particles size, distribution and number by nanoparticle tracking analysis (NTA); for morphology by electron microscopy (EM); expression of specific proteins by western blotting, enzyme-linked immunosorbent assay (ELISA), or flow cytometry; and RNAs by PCR [141–143]. However, the low expression of exosomal markers and the heterogeneity of exosomes limits their sensitivity and selectivity which restricts the application of these technologies [144]. These techniques produce results on the bulk sample rather than particular extracellular vesicles. Furthermore, these methods cannot indicate correlations of biomarkers in extracellular vesicles. Therefore, novel technologies capable of analyzing individual extracellular vesicles are needed to overcome these limitations [145]. Single extracellular vesicle analysis increases the sensitivity and improves the specificity by measuring the levels of specific cancer markers on extracellular vesicles or by identifying the exclusive subpopulations of circulating extracellular vesicles. Multiple single extracellular vesicle analysis techniques are already at the prototype stage. Extracellular vesicles are labelled in the solution phase in nearly all techniques as it minimizes the loss of circulating extracellular vesicles [146].

Exosomes derived from human ovarian epithelial cells (HOSEPiC) and ovarian cancer (ES-2, OVCAR3, and IGROV1) cell lines were characterized using electron microscopy (EM), dynamic light scattering (DLS), and NTA to study the difference between normal and malignant cancer-derived exosomes [147]. The total number of exosomes released from ovarian cancer cell lines is higher than that from normal cells. The exosomes derived from OVCAR3 showed the highest number of EpCAM-positive cells, while IGROV1 represented the least number of EpCAM-positive exosomes. The characteristics of exosomes were cell-type-specific, and NTA is a useful and efficient method to study particle size and concentration of exosomes in ovarian cancer.

Exosomes isolated from the blood of ovarian cancer patients using SEC are best characterized using NTA, compared with other conventional analysis methods like DLS, EM, and submicron particle analysis, as it represents a narrow size distribution and defines the concentration [148]. Clinical specimens consist of vesicles from diverse cellular origins. Therefore, it is crucial to identify their cell of origin, biological function, and the molecules expressed on their surface. The NTA fluorescence mode helps distinguish exosomes possessing specific markers. Surface-enhanced Raman spectroscopy (SERS) is another technique that was used by different researchers to analyze extracellular vesicles derived from ovarian cancer [149–152]. Multiple ovarian cancer cell lines (OVCAR 3, OV 90, EOC 6, and EOC 18)-derived extracellular vesicles were analyzed using SERS, and the major compositional difference was determined using principal component analysis [149]. Interestingly, machine learning based on logistic regression distinguishes ovarian cancer cell line-derived extracellular vesicles from normal cell-derived extracellular vesicles and high-grade ovarian cancer-derived extracellular vesicles from low-grade ovarian cancer-derived extracellular vesicles with approximately 99% accuracy, sensitivity, and specificity. A nanobowl SERS substrate coated with silver to analyze SKOV3-derived exosomes proves that the system characterizes exosomes at the molecular level [150]. A hybrid nanoplasmonic scaffold discriminates individual malignancies from each other and from controls using ovarian and endometrial cancer patients’ serum-derived extracellular vesicles. However, further standardization, validation, and cost effectiveness are required for clinical translation [151]. Thiolated LXY30 peptide-conjugated silver nanoparticles capture SKOV3-derived exosomes and with the help of SERS; the isolated exosomes were found pure, as multiple peaks associated with SKOV3 exosomes were present in SERS spectra, while these were absent in exosomes derived from Jurkat cells [152].

Different microfluidic platforms were developed that offer advantages in exosome isolation, proteomic and genomic analyses, and quantitative biology. These methods require a low amount of sample and offer simple processing, which makes their clinical translation practical [153]. Microfluidics-based platforms developed to characterize ovarian cancer-derived exosomes demonstrate promise for the prediction of ovarian cancer in clinical utilities. For example, a nanoplasmonic exosome assay is used to quantitatively analyze label-free exosomes [154]. The developed assay potentially recognizes exosomes derived from ascites of ovarian cancer patients by sensing CD24 and EpCAM. This approach is highly sensitive and provides label-free analysis with continuous real-time monitoring of exosome-specific molecular markers, unlike conventional techniques. The same laboratory that produced the Exosearch chip for the isolation and characterization of ovarian cancer-derived exosomes [140], developed a nano-IMEX microfluidic platform with the help of nanostructured graphene oxide/polydopamine for exosome analysis. The designed platform improves the immune-capture efficiency for exosomes and depletes non-specific exosome adsorption. The device distinguishes ovarian cancer patients from healthy controls, and requires only 2 µL of plasma without sample processing. The chip efficiently analyzes exosomes for the non-invasive diagnosis of disease and precision therapy of ovarian cancer [155]. A portable, eight-channel device was designed with an integrated magneto-electrochemical feature to immunomagnetically capture and characterize exosomes from the plasma of ovarian cancer patients [156]. The design is highly sensitive, capable of detecting cell-specific exosomes, provides sensor miniaturization, and high-throughput measurements. The sensor simultaneously detects multiple protein markers within an hour using 10 µL of samples per marker. This completely outclasses conventional methods in terms of speed and sensitivity. Furthermore, the device can be used for real-time monitoring of exosomal markers (CD24 and EpCAM) in the plasma of ovarian cancer patients pre-and-post drug treatment. The patients that did not respond to the treatment have elevated EpCAM and CD24 expression levels compared with patients who responded to the therapy. This device has slightly less sensitivity and throughput compared with iMEX, although it is less complex without nanofabrication. Therefore, it is a cost-effective portable platform for the on-site detection of exosomes. The Exocounter is a device that captures single exosomes in the groove of an antibody-coated optical disc labeled with antibody-conjugated magnetic beads, followed by counting using an optical disk drive to quantify the exosomes in the sera of ovarian cancer patients [157]. This device shows that the number of HER2-positive exosomes is elevated in the sera of ovarian cancer patients compared with those of healthy controls or non-cancer patients. The device is highly sensitive and represents linearity, unlike other conventional techniques. Hence, this approach is appropriate for liquid biopsies of exosomal biomarkers for ovarian cancer diagnosis. In addition to the above mentioned techniques, additional highly sensitive techniques such as localized surface plasmon resonance (LSPR), atomic force microscopy (AFM) [158–161], Imagestream [162], and droplet digital PCR [163] have been developed that can also be used for EV analysis in ovarian cancer.

In conclusion, all of the techniques developed for the isolation and detection of exosomes can potentially be used for diagnosing ovarian cancer and monitor cancer progression. However, some of these techniques will require further testing and validation using using a large cohort of ovarian cancer samples prior to their application in the clinic.

## Exosomes as biomarkers

The frequent late diagnosis of ovarian cancer at an advanced stage is considered a major reason for its high mortality rate [164]. Current techniques for the diagnosis of ovarian cancer include determining the carbohydrate antigen 125 (CA125) levels in the blood serum, transvaginal ultrasound, or physical examination, all of which have limited sensitivity and low specificity [15]. The level of the CA125 biomarker is generally not elevated early in ovarian cancer, and not all ovarian cancer patients have elevated levels. Poor specificity is a concern even in advanced disease, as CA125 levels can be elevated by other pathological conditions such as pelvic inflammation, breast cancer, and endometriosis [165]. The analysis of CA125 levels and transvaginal ultrasound in approximately 70,000 ovarian cancer patients showed that these methods had no impact on mortality rates; these findings should eliminate unnecessary surgeries due to false-positives [166]. Early diagnosis and subtype confirmation are essential for clinicians to design an appropriate treatment schema; therefore, there is an urgent need to identify novel biomarkers and methods for the early diagnosis and/or subtyping of ovarian cancer.

Cancer-related exosomes have garnered increasing attraction from researchers as possible cancer biomarkers owing to their presence in almost all body fluids and their specific distinctive features based on the cell of origin, which profoundly differ from the features of non-cancer exosomes [167]. These characteristics emphasize the potential diagnostic and prognostic worth of exosomes. Exosomes offer several advantages as a diagnostic marker. For example, their ubiquitous presence in almost all body fluids, including plasma, makes them a potential noninvasive alternative to biopsies. Moreover, exosomes are stable for months or years under specific storage conditions. Additionally, tumor-specific exosomal cargoes represent a precise association with tumor stages and prognosis [79]. The diagnostic worth of exosomal contents is confirmed using preclinical and clinical samples (Table 1 and described below).

**Table 1:** Preclinical and clinical studies demonstrating exosomal molecules as prospective biomarkers of ovarian cancer

**Molecule category**	**Molecule**	**Exosome Source**	**Subject**	**Reference**
**Preclinical studies**				
Protein	MRP2, ATP 7A, and ATP 7B	Cell culture medium	Ovarian carcinoma cells 2008 cells, 2008/C13*5.25 cells	[60, 94]
	Annexin A3	Cell culture medium and serum	Epithelial ovarian cancer cell lines, 50 ovarian cancer patients, and 30 healthy volunteer	[64, 92]
	Plasma gelsolin	Cell culture medium	Chemosensitive and chemoresistant cancer cell lines	[87, 168]
	Transketolase, glucose-6-phosphate dehydrogenase, and transaldolase	Cell culture medium	OVCA429 cell line	[94, 169]
	CEA, MUC16, MSLN, and WFDC2	Cell culture medium	SKOV3, OVCAR3, OVCAR5, and OVCAR433	[92, 170]
	Heat shock proteins like HSPB5, HSPB6, HSPB8	Cell culture medium	A2780, SKOV3, and cisplatin-resistant variants of both	[171, 172]
	Sialoglycoprotein galectin-3-binding-protein	Cell culture medium	OVMz cells	[168, 173]
	EGFR, HER2, CA125, FRα, CD24, EpCAM, CD 9, CD63	Cell culture medium	SKOV3 cell line	[169, 174]
Lipid	1227 lipids, 1433 proteins Lipids such as ChE, ZyE, Proteins such as lipoprotein lipase and collagen type V alpha 2	Cell culture medium	SKOV3 and HOSEPiC cell lines	[175]
miRNA	143 exosomal miRNAs	Cell culture medium	MCAS, HAC-2, TOV-112D, HRA, and OVAS cell lines	[176]
**Clinical studies**				
Protein	CD24 and EpCAM	Ascites	Cell lines and 16 ovarian carcinoma patients	[60]
	Claudin-4	Plasma	I63 ovarian cancer patients and 50 healthy volunteers	[64, 177]
	TGF-β1, MAGE 3, and MAGE 6	Plasma	22 ovarian cancer patients, 10 benign tumor patients, and 10 healthy volunteers	[87]
	CD24	Plasma	21 serous ovarian cancer patients and 8 healthy volunteers	[170, 171]
	CRABP2, SPP1, and TNFAIP6	Blood and sera of ovarian carcinoma patients	Ovarian cancer patients and healthy individuals	[177, 178]
	Soluble form of activated leukocyte cell adhesion molecule	Sera and ascites of ovarian cancer patients	Ovarian cancer patients and healthy controls	[178, 179]
	Soluble E-cadherin	Ascites or peritoneal fluid	35 ovarian cancer patients, 11 other cancer patients, and 6 non-cancerous (benign) patients	[172, 179]
	A total of 294 proteins identified, from which 225 proteins were present in all samples	Plasma	3 ovarian cancer patients and 6 health individuals for proteomic analysis. 40 epithelial ovarian cancer patients and 40 healthy volunteers for cohort study	[180]
Lipid	Phosphatidylserine	Plasma	34 suspected ovarian cancer patients and 10 healthy controls	[129, 173]
Nucleic acid				[174]
miRNA	miR-222-3p	Serum	6 epithelial ovarian cancer patients and 6 healthy volunteers	[75, 175]
	miR-200a-3p, miR-766-3p, miR-26a-5p, miR-142-3p, let-7d-5p, miR-130b-3p, miR-374a-5p, and miR-328-3p	Serum	155 ovarian cancer patients, 8 borderline patients, 43 benign tumor patients, and 63 healthy control	[129, 181]
	miR-21, miR-141, miR-200a, miR-200b, miR-200c, miR-203, miR-205, and miR-214	Serum	A total of 50 patients with serous papillary adenocarcinoma of the ovary (10 patients each for stage I, II, and IV and 20 patients for stage III)	[123]
	miR-21, miR-100, miR-220b, miR-320, miR-16, miR-93, miR-126, and miR-223	Plasma	A total of 143 participants: 106 diagnosed with epithelial ovarian cancer, 8 diagnosed with ovarian cystadenoma, and 29 healthy individuals	[75, 182]
	miR-1307 and miR-375	Serum	50 ovarian cancer patients, benign ovarian tumor patients, and healthy controls each	[181, 183]
	miR-34a	Plasma	58 epithelial ovarian cancer patients	[123, 184]
	miR-200c, miR-145, and miR-93	Serum	A total of 68 participants: 39 patients with high-grade serous ovarian carcinoma, 10 borderline patients, 10 benign disease patients, and 9 high-grade serous ovarian carcinoma patients	[182, 185]
	miR-200b, miR-200c, miR-200a, and miR-373	Serum	163 epithelial ovarian cancer patients, 20 benign tumor patients, and 32 healthy women	[183, 186]
	miR-146b-5p	Serum	5 epithelial ovarian cancer patients and 5 healthy controls	[184, 187]
	miR-92a	Urine	Ovarian cancer patients and healthy individuals	[185, 188]
	miR-30a-5p	Urine	A total of 95 participants: 39 ovarian serous adenocarcinoma patients, 26 benign disease patients, and 30 healthy individuals	[186, 189]
	miRNA-205	Plasma	36 ovarian cancer patients, 31 benign disease patients, and 32 healthy individuals	[187, 190]
	miR-4732-5p	Plasma	34 epithelial ovarian cancer patients and 21 healthy volunteers	[188, 191]
	miR-1290	Serum	71 epithelial ovarian cancer patients and 13 benign ovarian tumor patients	[189, 192]
	miR-1260a, miR-7977, miR-192-5p	Plasma	Epithelial ovarian cancer patients and healthy volunteers	[190, 193]
Circular RNA	Circ-0001068	Serum	95 ovarian cancer patients and 53 healthy controls	[192, 194]
	CircFoxp1	Serum	112 epithelial ovarian cancer patients and 82 healthy volunteers	[193, 195]
Long noncoding RNAs (lncRNAs)	MALAT1	Serum	60 epithelial ovarian cancer patients and healthy volunteers	[176, 196]
	UCA1	Serum	A total of 56 ovarian cancer patients: 32 cisplatin-resistant and 24 cisplatin-sensitive	[194, 197]
DNA	mtDNA	Plasma	24 serous epithelial ovarian cancer patients and healthy volunteers each	[195, 198]

### Preclinical samples

Exosomes derived from different ovarian cancer cell lines are generally collected and analyzed for specific marker expression in preclinical studies. The different proteins, lipids, or nucleic acids in the ovarian cancer cell-derived exosomes, can act as a prospective biomarker for the early detection of ovarian cancer through their altered expression. The activities of main regulatory enzymes of the pentose phosphate pathway (transketolase, glucose-6-phosphate dehydrogenase, and transaldolase) are increased in exosomes derived from two late-stage ovarian cancer cell lines: OVCA429 and HO8910PM [199]. Levels of plasma gelsolin that is released through exosomes are increased in chemoresistant ovarian cancer cells compared with those of chemosensitive cells [168]. This correlates with poor overall survival and relapse-free survival in ovarian cancer patients and shows that the exosomal transfer of plasma gelsolin is a marker for chemoresistance in ovarian cancer cells. Exosomes derived from chemoresistant cells show high expression of various resistance-related proteins, including MRP2, Annexin A3, ATP 7A, and ATP 7B; this clearly indicates the potential of exosomes to predict the efficacy of chemotherapy in ovarian cancer patients [92, 94]. The repression of N-glycosylation leads to alterations in the levels of exosome components and reduces the expression of various glycoproteins in ovarian cancer-derived exosomes [173]. This demonstrates the possibility of using exosome glycosignatures as biomarkers for ovarian cancer.

Few lipids have also been identified as potential biomarkers based on the considerable differences in various lipid species in exosomes derived from ovarian cancer cells (SKOV3) and ovarian surface epithelial cells (HOSEPiC) [175]. SKOV3-derived exosomes exhibit greater expression of ChE and ZyE lipids and produce a greater amount of lipoprotein lipase and collagen type V alpha 2 chain than do exosomes derived from HOSEPiCs. This highlights the possible role of exosomal lipids in the early detection of ovarian cancer.

Of the various exosomal payloads with diagnostic potential, RNAs are distinct, as free RNA is rapidly degraded in blood, whereas exosomal RNAs are protected from degradation [115]. Previous reports suggest that unusual miRNA expression in tumor tissue samples has diagnostic and prognostic potential in ovarian cancer. More recently, miRNA profiling of tumor-derived exosomes from patient plasma has shown clinical applicability of exosomal miRNAs as a circulating biomarkers [200]. Numerous studies emphasize the distinctive miRNA pattern in ovarian cancer patient-derived exosomes, and consistently show that exosomal miRNA is a promising diagnostic material in ovarian cancer [123, 181–183]. The miRNA composition of exosomes derived from ovarian cancer cell lines (CCCO, HRA, TOV-112D, HAC-2, OVAS, and MCAS) show 143 miRNAs with ≥ 1.5-fold elevated expression compared with those of normal cell (HOSE)-derived exosomes [176]. This suggests that the altered miRNAs can be validated as biomarkers for the progression of ovarian cancer.

It is evident from the study results described above that analysis of exosomal contents as potential biomarkers hold promise for ovarian cancer. However, it is worth mentioning that the histopathological origin of some of the widely used cell line models do not necessarily reflect the different subtypes of ovarian cancer. Hence the importance of using well-characterized cell lines as models for the particular subtype of ovarian cancer studied has been frequently emphasized [18].

### Clinical specimens

Exosomes collected from different biological fluids and tissue samples are tested as a source for prospective biomarkers for ovarian cancer diagnosis and prognosis prediction. Expression of TGFβ1, melanoma antigen (MAGE) 3, and 6 is upregulated in ovarian cancer derived-exosomes compared with that in exosomes derived from benign ovarian lesions (serous cysts) or normal cells suggesting that these proteins could be biomarkers to distinguish malignant and benign tumors [87]. Overexpression of CD24 and EpCAM in ovarian cancer cells is associated with poor prognosis and drug resistance. Both proteins are detected in exosomes from ovarian cancer cell lines and exosomes from malignant ascites. Analysis of a combination of CD24 and EpCAM with CA125 as tumor-dervied exosomal markers improved the accuracy for early diagnosis of ovarian cancer [140]. Claudin 4 which is overexpressed in ovarian cancer and associated with poor survival was detected in the exosomes from 32 of 63 (50.8%) ovarian cancer patients but in only 1 of 50 (2%) healthy individuals [64]. Therefore, claudin-4 is a potential biomarker for ovarian cancer detection. Detection of a panel of biomarkers (EGFR, CA125, EGFR2, folate receptor α, CD9, CD24, and EpCAM) in circulating exosomes not only identified ovarian cancer patients from control subjects but also distinguished between early- and advanced- stage ovarian cancer [174]. Proteomic profiling of exosomes from the plasma of epithelial ovarian cancer patients by liquid chromatography tandem mass spectrometry (LC–MS/MS) with tandem mass tagging (TMT) identified set of proteins associated with coagulation pathway that was not observed in exosomes from healthy individuals [180]. Additionally, set of four genes showed promise as diagnostic markers and two genes as prognostic markers in ovarian cancer. Additionally, higher levels of proteins in ovarian cancer-derived exosomes that have diagnostic potential include CD24, CRABP2, MUC16, MSLN, soluble form of activated leukocyte cell adhesion molecule, and soluble E-cadherin [171, 177–179]. Levels of small heat shock proteins have also been reported to be elevated in serum and peritoneal fluid-derived exosomes from ovarian cancer patients that correlated with immune cytotoxic markers; however, further research is required to confirm their diagnostic potential [172]. Besides proteins, phospholipids on exosomes have also been investigated as biomarkers in cancer diagnosis and therapy. There is a notably greater amount of phosphatidylserine in exosomes derived from the plasma of ovarian cancer patients than in those from healthy individuals [129]. Furthermore, phosphatidylserine levels are higher in patients with malignant tumors than in patients with benign lesions. This provides proof of concept for the diagnostic potential of phosphatidylserine in ovarian cancer.

miRNAs enriched in exosomes have also been investigated as cancer biomarkers in the diagnosis of ovarian cancer and in differentiating from benign diseases. Several studies document differential and overexpression of miRNAs in ovarian cancer than in benign disease and healthy individuals. Exosomes isolated from circulating tumor exosomes recovered from the sera of ovarian cancer patients showed differential and distinct expression of eight miRNAs (miR-21, miR-141, miR-200a, miR-200b, miR-200c, miR-203, miR-205, and miR-214) compared to those with benign disease and not detectable in healthy individuals [123]. Additionally, the cellular and exosomal levels of the eight miRNAs in ovarian cancer patients matched suggesting that the exosomes-based miRNAs hold the potential to be used as diagnostic markers for screening and identifying ovarian cancer patients from those with benign disease and a replacement for biopsy. Serum-derived exosomal miR-34a expression is markedly higher in early-stage ovarian cancer patients than in advanced-stage patients, in non-metastatic patients than in those with lymph node metastasis, and in non-relapsed patients than in those who relapsed [184]. Exosomal miR-34a expression was also examined across histological types and found to be higher in early stage ovarian cancer compared to late stage ovarian cancer in non-serous carcinoma (clear-cell, endometrioid, and mucinous carcinoma). However, no substantial variation in exosomal miR-34a levels was seen between early and late stages of serous carcinoma. These findings highlight the potential of exosomal miR-34a as a biomarker for detection of ovarian cancer subtype and differentiating between early and late stage of the disease. Analysis of a set of miRNAs that are overexpressed in ovarian cancer showed miR-200c, miR-145, and miR-93 is elevated in serum-derived exosomes from ovarian cancer patients compared to patients with benign disease and borderline ovarian cancer [185]. Furthermore, miR-200c but not miR-145 was significantly increased in high grade serous carcinoma compared to non-high grade serous carcinoma patients. However, miR-145 showed the highest sensitivity in discriminating ovarian cancer from benign disease holding it to be promising biomarker for pre-operative diagnosis. High levels of miR-21, miR-100, miR-200b, and miR-320 have been reported in plasma-derived exosomes from epithelial ovarian cancer patients than in exosomes from healthy controls [182]. From the four miRNAs identified, only miR-21 was found to be significantly less in exosomes from patients with serous EOC compared to patients with other histological subtypes. Increased miR-200b expression correlated with CA125 levels and overall poor survival. Significantly higher expression of miR-200b and miR-200c is observed in stage III-IV patients (including those with lymph node metastasis) than in stage I-II patients [186]. miR-146b-5p expression is found to be higher in exosomes from epithelial ovarian cancer patients compared with that in the healthy control group [187]. Recently, exosomes in urine have drawn researcher’s attention as they represent easily accessible samples [32]. Urine-derived exosomes had higher levels of miR-92a expression ovarian cancer patients [188]. Similarly, increased miR-30a-5p levels in exosomes from urine samples of ovarian serous adenocarcinoma patients compared with that in healthy individuals was reported [189]. However, the diagnostic ability of urinary exosomal miRNAs might be limited owing to their dependence on urine fractions and their vulnerability to the external environment. Additional exosomal miRNAs that have been reported to serve as potential diagnostic markers for ovarian cancer include miRNA-205, miR-4732-5p, miR-1290, miR-1260a, miR-7977, and miR-192-5p [190–193]. These study results further demonstrate that exosomal miRNA is a powerful diagnostic marker for ovarian cancer.

Other exosomal nucleic acids including circRNAs, such as Circ-0001068 [194] and CircFoxp1 [195]; lncRNAs, such as MALAT1 [196] and UCA1 [197]; and mitochondrial DNA (mtDNA) [198] demonstrate potential as biomarkers for the early detection of ovarian cancer. Currently, there are two ongoing clinical trials to assess the diagnostic potential of exosomal cargo. One study analyzes the expression of miRNAs and lncRNAs in exosomes from patients with high-grade serous ovarian cancer and benign gynecologic diseases (NCT03738319). The second study focuses on the diagnostic and prognostic impact of circulating tumor (ct) DNA and exosomes in digestive, gynecological, and breast cancers (NCT04530890).

Despite the exciting findings in preclinical and clinical studies, application of exosomal contents as cancer diagnostic and prognostic markers remain investigational and need to be compared with additional tumor-derived biosources for efficiency as a liquid biopsy [201]. Thus, extensive research is necessary to reveal the practicability of exosomal cargo for ovarian cancer diagnosis and prognosis prediction.

## Exosomes in therapeutics

There is a burgeoning interest in exploring the use of exosomes in the therapeutic arena. The likely therapeutic approaches include restricting the secretion of tumor-derived exosomes, encapsulating various cargoes such as drugs or nucleic acids within exosomes, or augmenting immunotherapy based on increased information about the role of exosomes in cancer.

### Targeting exosomes as a cancer treatment

Exosomes play a vital role in ovarian cancer progression and metastasis. Therefore, targeting exosomes may have fruitful clinical implications for the treatment of ovarian cancer [65, 201]. This can be achieved by inhibiting the production and secretion of exosomes from cancer cells or through the removal of exosomes from peripheral circulation.

#### Direct targeting of exosomes

There are no clinically available drugs to efficiently remove tumor-derived exosomes from circulation in cancer patients [65]. However, there are multiple options available to target or exploit exosomes for ovarian cancer therapy [174]. High-throughput screening of 4580 compounds identified five molecules (ketoconazole, climbazole, tipifarnib, triadimenol, and neticonazole) that inhibit exosome biogenesis and secretion in C4-2B prostate cancer bone metastasizing cells [202]. However, additional research is needed to confirm their efficiency in vivo. Various other small molecules have also been found to inhibit exosome production. For example, sphingomyelinase inhibitor, GW4869, leads to reduced exosome formation in Oli-neu cells by inhibiting ceramide, an important molecule for exosome production [203]. This is supported by a reduction in lung metastasis following an inhibition of exosome secretion; this result is partly reversed by injecting tumor-derived exosomes into mice. Exosome secretion is mediated via H^+^/Na^+^ and Na^+^/Ca^2+^ channels. Blocking these channels using dimethyl amiloride reduced the exosomes production and secretion and reduced the immunosuppressive action of myeloid-derived suppressor cells (MDSCs), which elevated the anticancer activity of cyclophosphamide in three distinct mouse tumor models [204]. Rab family proteins (Rab27a and Rab27b) play an important role in exosome release and silencing these proteins reduced exosome secretion in HeLa cells [205]. Inhibiting exosome secretion with GW4869 and exosome uptake using 5-(N-ethyl-N-isopropyl)-amiloride suppressed tumor growth in an ES-2 xenograft mouse model of ovarian cancer, and Rab27a knockdown inhibits tumor growth and increases survival in mice [206]. Phosphatidylserine is reported to be overexpressed on the surface of ovarian cancer-derived exosomes. (ZnDPA)_6_-DP-15 K referred to as Exoblock is a novel multivalent phosphatidylserine binder that has high binding avidity to exosomes. Administration of Exoblock in vivo against an ovarian tumor-based omental tumor xenograft model and a melanoma-based xenomimetic (X-) mouse model reduces phosphatidylserine positive exosomes and increases the amount and function of CD4 and CD8 T cells leading to the suppression of tumor recurrence of melanoma and progression and metastasis of ovarian cancer [207].

#### Exosome elimination from peripheral blood circulation

Exosomes are closely associated with tumor invasion and metastasis. Therefore, their removal from peripheral circulation may be of a therapeutic benefit. Aethlon Medical, USA, has developed a proprietary lectin-based affinity column-based filtration system called the “Hemopurifier” that depletes tumor-derived exosomes from the blood [208]. The Hemopurifier is a non-pharmacological intervention that is free from drug-related toxicities. A safety study of the device was recently completed, although the results are unavailable to the public (NCT02215902). The Hemopurifier is analyzed in clinical trials for the treatment of SARS-CoV-2 (COVID-19) to clear the virus and improve the outcomes of infected patients (NCT04595903). Furthermore, the device is used in another trial in combination with pembrolizumab (Keytruda) as a first line treatment to clear immunosuppressive exosomes in patients with advanced and/or metastatic head and neck squamous cell carcinoma (NCT04453046). The device has not been used in ovarian cancer patients. A positive outcome of ongoing clinical trials will help direct its use in different cancers, including ovarian cancer.

### Targeted drug delivery using exosomes

**Fig. 5:**
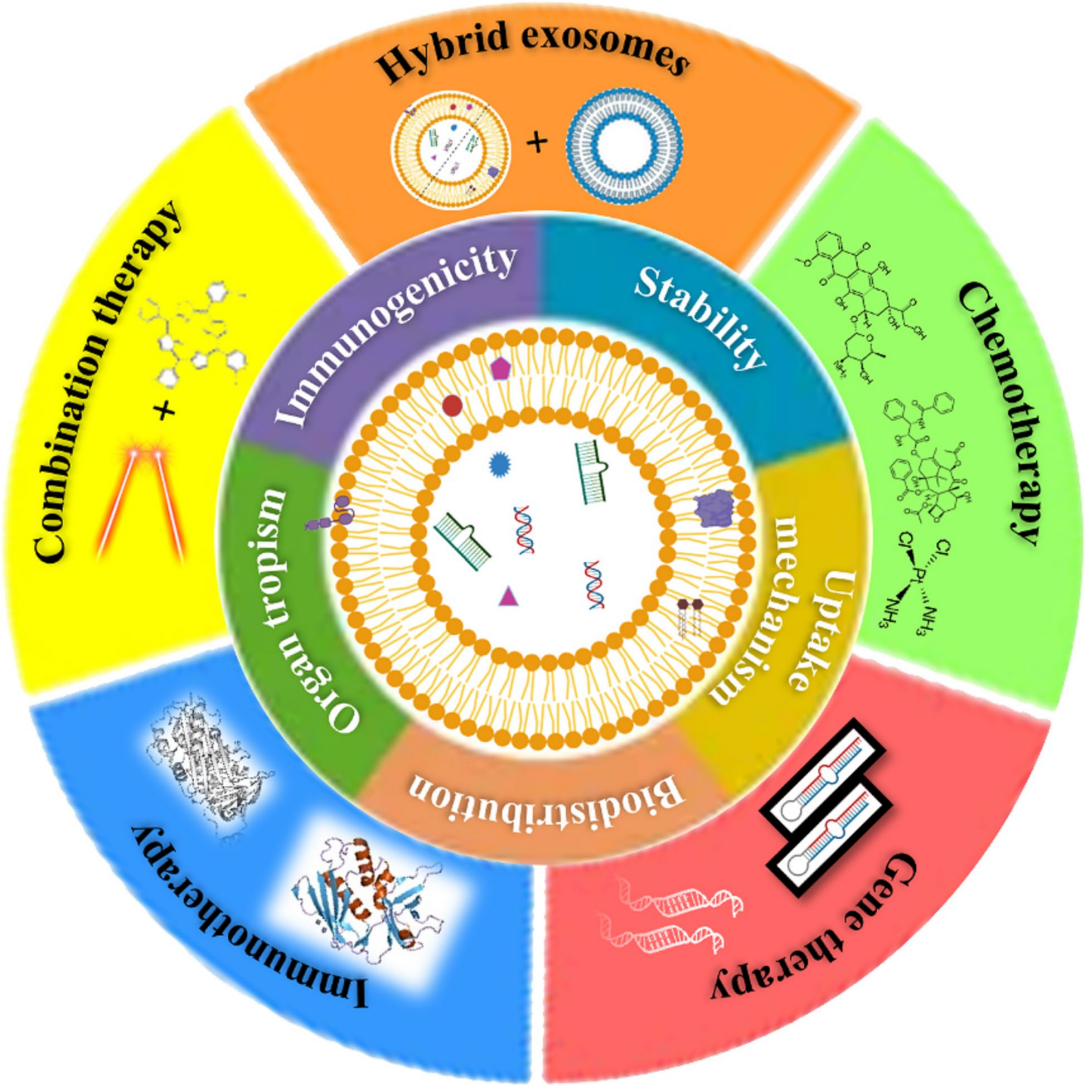
Exosomes as nanocarriers offers several benefits and numerous exosomes-based therapeutics are being applied for treating ovarian cancer. Image created with BioRender.com

Improved drug delivery systems are a pressing need in cancer therapy to increase therapeutic efficacy, reduce toxicity burdens, and potentially reduce associated costs. Exosomes are viewed as particularly promising candidate drug carriers based on their ability to overcome existing pharmacokinetic complications. Furthermore, their systemic stability, biocompatibility, immunocompatibility, cell uptake mechanism, and biodistribution properties make them ideal candidates for anticancer drug delivery. They can enhance the therapeutic outcomes by specifically targeting cancer cells and reducing undesired side effects [209]. Table 2 presents comparisons of some of the characteristics of exosomes with other cell-based carriers, while, Fig. 5 highlights various advantages of exosomes as drug carrier and delivery approaches in attempts to treat ovarian cancer.

**Table 2:** Characteristics of exosomes and other biological carriers explored for drug delivery [210–213]

Properties	Exosomes	RBCs	Leukocyte	Platelets
Amount (/mL, blood or plasma)	~ 10^10^	4.2-6.0 × 10^9^	4.5-11.0 × 10^6^	1.5-4.5 × 10^8^
Size (µm)	30–100 × 10^–3^	~ 7	7–15	1–4
Immunocompatibility	Immunocompatible	Immunocompatible (autologous immunocompatibility)	Immunocompatible	Immunocompatible
Life span	A few minutes to hours	Long; 100 –120 days	A few days	7–10 days
Targeting ability	High	Low	High	High
Tumor homing ability	Able	Unable	Able	Able
Need for ex vivo preparations	Not required	Not required	Required	Required

#### Advantages of exosomes as drug carriers

##### Immunogenicity

Foreign particles are usually antigenic and prompt immune activation [214]. Therefore, allogenic and/or heterologous cell-derived exosomes may cause undesirable immune activation. However, blood or plasma transfusions with over a trillion exosomes from multiple sources without matching inter-patient human leukocyte antigen (HLA) do not generate immune responses in patients [215, 216]. Thus, exosomes derived from allogenic and/or heterologous cells may not be unduly immunogenic [209]. Despite other biological nanoparticles, such as erythrocytes, are deemed immunocompatible, they are largely immunocompatible in autologous administrations, therefore a lot of researchers are attempting to create universal RBCs from non-O group [210, 217]. Furthermore, in such cell-based carriers, generally cargo is loaded on their surface, and that may also contribute to immunogenicity [218].

A study to determine exosome toxicity and immunogenicity in mice by systemically administering HEK293T cell-derived exosomes three times a week for three weeks (total of 10 doses, 10^10^ exosomes per dose) found no significant differences in proinflammatory cytokine levels, biochemical parameters, or complete blood counts in the treatment group compared with those of the control group [219]. A vast number of microbes (3.8 × 10^13^) are present in the human body (the microbiome), and the membrane vesicles they secrete can enter the bloodstream; microbe-derived extracellular vesicles are usually present without undue effects [220]. Plant- and milk-derived exosomes do not generate any significant immune response [221–223]. Nevertheless, the addition of DNA to parental cells to generate engineered exosomes may alter the exosome composition. Hence, it is necessary to systematically evaluate the immunogenicity of engineered exosomes.

##### Stability

Exosomes generally exhibit good stability and integrity over a prolonged period of time. Urine samples stored at -20°C exhibit a significant loss of urinary exosomes; however, almost all exosomes (86%) are recovered from samples stored at -80°C [224]. Milk-derived exosomes in simulated gastrointestinal fluid are stable for 2 h [225]. Long-term storage of the same exosomes at -80 °C shows no changes in physical properties or percent drug loading over four weeks. Plasma-derived exosomal miRNAs have exceptional stability under various storage conditions [226]. Meanwhile, the size of exosomes from three different cell types (mesenchymal stem cells, HEK 293T cells, and endothelial colony-forming cells) stored at -20 °C did not change, even after multiple freeze–thaw or ultracentrifugation cycles [227]. Exosome-encapsulated nucleic acids are protected from degradation by RNases [228]. Thus, exosomal stability is an important advantage for drug delivery and cancer therapy.

##### Biodistribution and pharmacokinetics

It is necessary to understand the pharmacokinetics of exosomes to fully understand their biological function and apply them therapeutically. The first step involves evaluating their biodistribution [229]. Intravenously injected B16F10 melanoma cancer cell-derived exosomes mainly accumulate in the bone marrow, lungs, liver, and spleen of mice [230]. The administration of mesenchymal stem cell-derived exosomes to normal mice results in their accumulation in the liver and spleen [231]. Orally administered exosomes are equally distributed within the lungs, kidney, ovaries, pancreas, liver, colon, spleen, and brain, while intravenously administered exosomes primarily accumulate in the liver [223]. This reflects the importance of the route of administration. Likewise, intravenous administration of exosomes derived from bone marrow-derived dendritic cells, murine B16F10 cells, HEK 293T cells, and C2C12 myoblasts resulted in preferential accumulation in the spleen, liver, lungs, and gastrointestinal tract. These data support exosomes derived from various cellular origins and route of administration influence their organ specific homing abilities in vivo [232].

Multiple studies have assessed the pharmacokinetic properties of exosomes. While fluorescent labelling of exosomes reveals adequate in vivo accumulation, the low sensitivity and release of the free dye limit the utility of fluorescent dyes in assessing the pharmacokinetic profile of exosomes [209, 229]. A better approach incorporates luciferase, as bioluminescence detection offers greater sensitivity [233]. Murine melanoma B16-BL6-derived exosomes are rapidly eliminated after systemic administration to mice (blood half-life = two minutes), which is was determined using *Gaussia* luciferase and lactadherin fusion protein [234]. The same method determined that the half-life of multiple cell line-derived exosomes in mice is between two and four minutes [235, 236]. However, recent studies showed that exosomes have unique features that prolong their biological half-life. The expression of CD47 in mesenchymal stem cell-derived exosomes protects exosomes from recognition by phagocytic cells and increases their circulation time in vivo [237]. Intraperitoneal administration of mesenchymal stem cell-derived exosomes engineered to contain siRNAs targeting K-RAS mutants in pancreatic cancer show exosomes in systemic circulation even after 24 h. Thus, altering the exosomal surface to escape phagocytic cells and the reticuloendothelial system may increase the biological half-life, and exosomes from mesenchymal stem cells may be applicable for antitumor therapy.

##### Cellular uptake mechanism

The internalization of exosomes occurs by different mechanisms including endocytosis, micropinocytosis, phagocytosis, and fusion. The internalization of exosomes by dendritic cells occurs via clathrin-mediated endocytosis [238], while exosomes are taken up by phagocytes via phagocytosis [239]. Microglia take up exosomes via micropinocytosis, and the exosomal surface lipids and phosphatidylserine activate the process [240]. Exosomes fuse with the membrane of melanoma cells to deliver loaded molecules [241]. The interaction of SKOV3-derived exosomes with ovarian cancer cells confirms that exosome internalization occurs through an energy-dependent endocytic pathway [242]. These discrete cellular internalization pathways support the potential therapeutic advantage of exosomes as a drug carrier. However, thorough research is required to understand the cellular uptake of exosomes by specific cell types before developing novel exosome-based cancer therapeutics.

##### Cell tropism and tumor homing

Exosomes carry proteins and lipids that reflect their cell origin; therefore, they exhibit tropism toward their parental cells. This unique property eliminates the requirement for surface modification of the exosomes to achieve tumor-targeted delivery. The administration of exosomes derived from HT1080 or Hela cells to HT1080 tumor-bearing nude mice confirms tumor homing by the cancer-derived exosomes [243]. pH-sensitive exosomes derived from two different tumor cell lines (BT-474 and SK-N-MC) have tumor-homing ability [244]. Mesenchymal stem cell-derived exosomes display tumor-homing ability [245]; this makes exosomes ideal candidates for tumor-targeted drug delivery systems.

#### Drug loading into the exosomes

As exosomes possess natural ability to carry different molecules inside their lumen and deliver the cargo to target site, researchers have utilized them as a vehicle to carry anticancer therapeutics. The methods to load anticancer therapeutics within exosomes can be broadly classified into two categories: active or direct loading, and passive or indirect loading. Readers can find the detailed information about conventional drug loading techniques in the article published by Srivasatava et al. [31]. More recent advances in drug loading into exosomes include microfluidics-based strategies. Exo-Load, a microfluidic device, was reported to load up to 19.7% of DOX in glioma-derived exosomes, which was equivalent to electroporation and sonication procedures [246]. By changing the instrument fabrication to sigmoid type, loading efficiency of DOX was increased to 31.98% at 12.5 L/min injection flow rate. Paclitaxel which is hydrophilic was also effectively loaded into glioma-derived exosomes using the apparatus. Though microfluidics-based techniques can help increase drug loading into exosomes, there are very few pre-clinical and clinical reports proving their performance at this time. Therefore additional studies are needed to determine their drug loading efficiency and anticancer efficacy.

#### Exosomes as a therapeutic platform for ovarian cancer treatment

The percentage of drug cargo reaching the tumor after in vivo administration using several drug delivery strategies is low and inadequate to completely eradicate the tumor [247]. Exosomes display exceptional characteristics that make them an ideal vehicle for drug delivery. Therefore, they are being vigorously explored for the loading of anticancer drugs and/or biologics with a view to develop effective therapy against cancers, including ovarian cancer. Table 3 presents published studies using exosomes as nanocarriers to deliver therapeutic cargo in ovarian cancer.

**Table 3:** Exosome-mediated delivery approaches for the treatment of ovarian cancer

Therapeutic Class	Therapeutic	Source of exosome	In vitro/in vivo model	Outcome^a^	Reference
Chemotherapy	Doxorubicin	MDA-MB-231 and STOSE cells with or without CD-63 GFP	Syngeneic model of breast cancer and high-grade serous ovarian cancer	↓ Cardiotoxicity ↑ Efficacy and safety	[248]
	Cisplatin	Macrophage from the umbilical cord blood of patients	In vitro: Drug-resistant and -sensitive A2780 cells	↑ Cytotoxicity	[249]
	Cisplatin	Milk	Ovarian tumor xenograft model	↑ Anticancer activity	[250]
	Paclitaxel	Mesenchymal stromal cells, Human umbilical vein endothelial cells	MDA-hyb1 breast cancer cell-bearing NODscid mice	↑Cytotoxicity and tumor specificity	[251]
	Doxorubicin	ThP-1 cells	In vitro: 2D and 3D cell cultures of SKOV3 cells	↑ Cytotoxicity	[252]
	Berry anthocyanidins, Paclitaxel	Milk	A2780 tumor xenografts	↑ Antiproliferative activity	[253]
	Triptolide	SKOV3 cells	SKOV3 tumor xenografts	↓ Tumor cell proliferation and tumor growth	[254]
Gene therapy	miR-199a-3p	Omental fibroblasts of ovarian cancer patients (primary culture)	Ovarian cancer mouse model	↓ c-Met expression ↓Ovarian cancer cell proliferation and invasion	[255]
	miR-92b-3p	SKOV3 cells	Zebrafish and nude mouse model	↑ Antiangiogenic and anticancer activity Synergism with apatinib	[256]
	miR-146a	Mesenchymal stem cells	In vitro: SKOV3 cells, A2780 cells, and their chemoresistant mutants	↓ Chemoresistance ↓ Cell growth	[257]
	Cas9 and PARP-1 sgRNA (CRISPR/Cas9)	HEK293 and SKOV3 cells	SKOV3 xenograft mouse model	↑ Cisplatin sensitivity ↑Ovarian cancer apoptosis	[258]
	Human carbonyl reductase 1 DNA	Endometrial stromal cells	In vitro: TOV-21G, SKOV3 cell lines	↓ Cancer cell proliferation	[259]
Immunotherapy	Staphylococcal enterotoxin B	SKOV3 cells	In vitro: SKOV3 cell line	↑ Apoptosis rate ↑Caspase-9 and caspase-3 expression	[260]
	Ovalbumin-derived tumor antigen lysates	Dendritic cells	Metastatic ovarian cancer mouse model	↑ Tumor, peritoneal metastatic nodule, and liver accumulation	[261]
	Exosome priming with dendritic cells	Malignant ascites from ovarian cancer patients	In vitro: patient-derived ovarian cancer cells	↑T cell differentiation ↑Increased cytotoxicity	[82]
Combination therapy	Meta(tetrahydroxyphenyl)chlorin (immune active photosensitizer)	Mesenchymal stem cells	Peritoneal metastasis model of ovarian and colorectal origins	↑ Tumor specificity ↑Tumor-specific photodynamic cytotoxicity Anticancer immune response	[262]
	Cetuximab or trastuzumab scFv	CAR-T cells	MDA-MB-231 and HCC827 mouse xenograft models	↑ Cytotoxicity ↓ Tumor growth Safety proven in preclinical model of cytokine release syndrome	[263]
Hybrid exosome (Exosome + liposome fusion)	Triptolide and miR497	Cisplatin-resistant SKOV3-CDDP cells	Cisplatin-resistant SKOV3-CDDP tumor-bearing BALB/c-nu mice	Chemoresistance overcame Reactive oxygen species ↑M2 to M1 macrophage polarization ↑Tumor accumulation ↑ Anticancer activity	[264]
Surgical implant (M-trap)	NA	Ascites from ovarian cancer patients	Female SCID beige mice	Ovarian cancer cells disseminated to the peritoneal cavity trapped ↑ Mean survival time	[265]

^a^↑denotes upregulation or increase; ↓denotes downregulation or inhibition

##### Exosomal chemotherapy

Exosomes have been studied to deliver various synthetic and natural cargoes to improve the therapeutic efficacy in ovarian cancer. The delivery of doxorubicin by exosomes can overcome cardiotoxicity. This facilitates greater efficacy of doxorubicin against breast cancer and ovarian cancer in vivo [248]. Similarly, the treatment of drug-resistant and drug-sensitive ovarian cancer cells with cisplatin-loaded exosomes from umbilical cord macrophages significantly increases cytotoxicity [249]. The increase in cytotoxicity is much higher in drug-resistant cells, and the exosomes represent a potent carrier for treating drug-resistant cancers. It has been found that ovarian cancer cells (A2780 and A2780/DDP) exhibit greater integrin/CD29 receptor expression, and M1 macrophage derived exosomes contain highly expressed integrin/CD29 [266]. Exosome uptake by A2780/DDP cells was drastically reduced when CD29 was blocked. As a result, integrin/CD29 may be regarded as a targeting receptor for the mechanism of action of macrophage-derived exosomes against ovarian cancer cells. Milk-derived exosomes loaded with cisplatin increase anti-cancer activity in vitro and in vivo in an ovarian cancer xenograft model [250]. Exosomes deliver drugs to resistant cells via clathrin-independent endocytosis; this process improves anti-cancer activity, as it evades endosome trapping. Mesenchymal stem cells and human umbilical vein endothelial cell-derived exosomes loaded with paclitaxel have 80–90% cytotoxicity against the ovarian cancer cell line (SKOV3), lung cancer cell line (A549), and breast cancer cell line (MDA hyb1), while control exosomes are not significantly cytotoxic [251]. Exosomes and monocyte-derived exosome mimetics loaded with doxorubicin showed increased cytotoxicity compared to the free drug [252]. Milk-derived exosomes loaded with berry anthocyanidins significantly elevate antiproliferative activity and suppress the growth of ovarian cancer cells. A2780 xenograft mice treated with a combination of berry anthocyanidin and paclitaxel-loaded exosomes showed considerably increased anticancer activity; the authors concluded that milk-derived exosomes are an outstanding platform for drug delivery in ovarian cancer [253]. Similarly, triptolide-loaded SKOV3-derived exosomes profoundly inhibit tumor cell proliferation and tumor growth in Balb/c nude mice bearing SKOV3 tumors [254]. Together, these studies suggest that exosomes represent an efficient drug delivery vehicle, and exosomal drug delivery is an excellent candidate for treating ovarian cancer.

##### Exosomal gene therapy

Exosomal ncRNAs play a key role in ovarian cancer pathogenesis. Therefore, designing platforms that precisely target exosomal cargo holds significant promise for ovarian cancer therapy [34]. Exosomes from the omental fibroblasts of ovarian cancer patients loaded with miR-199a-3p restrict ovarian cancer cell proliferation and invasion by downregulating c-Met expression [255]. These exosomes dramatically reduced peritoneal dissemination in a mouse model of ovarian cancer [255]. Peptide-modified miR-92b-3p–transfected exosomes have greater anti-angiogenic and antitumor activity and are synergistic with apatinib in nude mouse and zebrafish models of ovarian cancer [256]. Treatment of drug-resistant SKOV3 cells and A2780 cells with mesenchymal stem cell-derived exosomal miR-146a inhibits cancer cell growth and drug resistance [257]. CRISPR/Cas9 directed genome editing of tumor-derived exosomes inhibit poly (ADP-ribose) polymerase-1 levels and enhance the sensitivity of the SKOV3 xenograft mouse model to cisplatin; exosomes loaded with PARP-1 sgR/Cas9 reduce the tumor size in vivo [258]. Meanwhile, carbonyl reductase 1-overexpressing exosomes significantly inhibit the proliferation of ovarian cancer cells [267]. Thus, exosomes can be successfully used as a carrier to deliver genes to treat ovarian cancer.

##### Exosomal immunotherapy

Immunotherapy is increasingly applied in cancer therapy, and exosomes can be utilized in cancer immunotherapy owing to their immunomodulatory characteristics [268]. Staphylococcal enterotoxin B anchored to SKOV3-derived tumor exosomes increase cytotoxicity and apoptosis upon treatment of SKOV3 cells [260]. Furthermore, caspase-9 and caspase-3 expression is upregulated, although there is no change in the expression of cancer-promoting genes. A dendritic cell-derived exosome-based vaccine for the treatment of ovarian cancer was prepared by pulsing dendritic cells with tumor antigen lysates and collecting the dendritic exosomes with ovalbumin on their surface [261]. Intraperitoneal injection of these exosomes in a mouse model of metastatic ovarian cancer showed superior accumulation of exosomes in tumors, peritoneal metastatic nodules, and the liver. These initial results show that dendritic cell-derived exosomes are a prospective candidate for metastatic ovarian cancer therapy. Further investigations are continuing to determine the ability of exosomes to generate antitumor immune activation and increase survival when combined with chemotherapy. Additionally, exosomes have immunotherapeutic potential, as they can transport various ovarian cancer antigens. For example, the transfer of antigen-containing tumor-derived exosomes to dendritic cells leads to cytotoxic T lymphocyte expansion and generates an anticancer response [269]. Similarly, the transfer of tumor-associated antigens from exosomes derived from metastatic ovarian carcinoma to dendritic cells increases their cytotoxicity [82]. However, there are limited reports testifying the use of exosomes in ovarian cancer immunotherapy, and more research is required, to explore the potential of exosomes as ovarian cancer immunotherapeutics.

##### Exosomal combination therapy

**Fig. 6:**
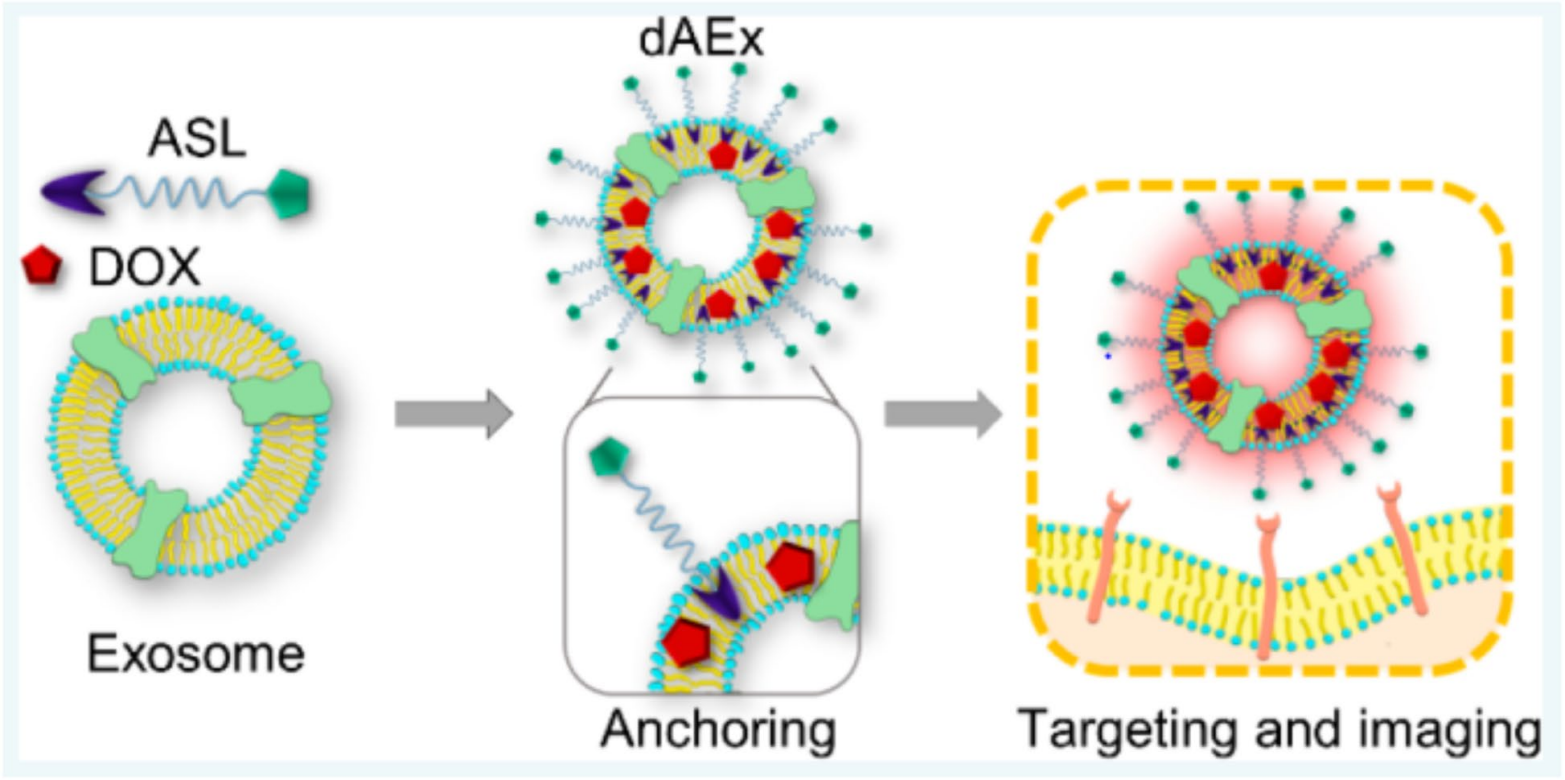
Exosome-based combination therapy for melanoma. Anchor (BODIPY)-spacer (PEG)-targeting ligand (cyclic RGD peptide) functionalized exosomes loaded with doxorubicin for melanoma therapy. Image reproduced with permission from Kang et al. [272] copyright (2020) American Chemical Society

Combination therapy is the centerpiece of cancer treatment and includes the combination of two or more therapeutic options [270]. Exosomes have been studied in combination therapy to combat ovarian cancer. Loading the immune active photosensitizer (meta(tetrahydroxyphenyl)chlorin) in mesenchymal stem cell-derived exosomes to target peritoneal metastasis triggers tumor-specific photodynamic cytotoxicity and generates an anticancer immune response. These results suggest tumor specificity of the exosomes, improved therapeutic efficacy, and decreased tumor progression in peritoneal metastasis of ovarian cancer [262]. CAR exosomes isolated from CAR-T cells loaded with cetuximab or trastuzumab scFv, respectively target and treat EGFR- or HER-2-positive breast and ovarian cancer [263]. These CAR exosomes were highly cytotoxic, significantly inhibit tumor growth in vivo, and are much safer than CAR-T therapy in a preclinical model of cytokine release syndrome [263]. Exosomes from expanded NK cells were shown to be preferentially taken up by SKOV3 cancer cells resulting in their kiling [271]. It was discovered that NK cells treated with expanded NK cell derived exosomes have more cytotoxicity than control NK cells. Transcriptional analysis revealed altered expression of various chemokine ligands on NK cells that likely contributed to the increased cytotoxicity. Additionally, a recent targeted therapy approach was used to engineer exosomes with a cyclic RGD peptide (referred to as ASL exosomes) capable of targeting α_V_β_3_integrin in melanoma models (Fig. 6) [272]. Doxorubicin was further loaded into these exosomes (dAExs). Treatment of mouse bearing B16F10 melanoma tumors with dAExs led to reduced tumor growth in mice. Additionally, the ASL modification allows for efficient imaging of tumor cells. A similar approach of exosome-based combinatorial therapy may benefit ovarian cancer. Researchers from Cardiff University, UK, provide a rationale for using chemo-immunotherapy with Toll-like receptor agonists such as poly[I]:poly[C12U] and tumor exosomes to treat advanced ovarian cancer, and clinical trials are planned [273, 274]. Results from this clinical study will provide more information on the efficacy of exosome-based combination therapy in humans.

##### Other approaches

Liposome-exosome hybrid nanoparticles were developed using a fusion technique to simultaneously deliver triptolide and miR497 in ovarian cancer [264]. These hybrid nanovesicles enhance the cytotoxicity and apoptosis of ovarian cancer cells in vitro, accumulate in tumors, and exhibit increased anticancer activity in vivo. Furthermore, they overcome cisplatin resistance in ovarian cancer cells. Exosomes derived from the ascites of ovarian cancer patients were embedded in a 3D scaffold and surgically implanted in female SCID Beige mice [265]. This scaffold was used to trap ovarian cancer cells that disseminate to the peritoneal cavity, and a remarkable increase in the mean survival time was observed in presence of the implant: 117.5 to 198.8 days. Removal of the implant increased the mean survival time to 309.4 days. These reports show that exosomes are either directly targeted or can be used to deliver therapeutic cargoes for the treatment of ovarian cancer. Another approach that holds promise for ovarian cancer treatment is the use of exosomes loaded with epigentic modulators in combination with chemotherapy, immunotherapy, and small molecule inhibitors [275].

## Safety and toxicity considerations

Exosomes have low immunogenicity because of their biological origin. There are many daily blood/plasma transfusions resulting in the administration of countless exosomes to patients without evident side effects. Exosomes are safer than other biological carriers (viral vectors or cell therapy), as they are non-mutagenic and cannot replicate. Thus, there are minimal regulatory concerns regarding toxicity or neoplasia development. Minimal to negligible toxicity is reported in in vivo exosomal therapeutic studies [276]. HEK293T cell-derived exosomes are non-toxic upon systemic administration to mice [219], while siRNA-engineered exosomes lack toxicity even after repeated administration in mice [237]. These studies and multiple other experiments prove that exosomes are safe. However, the complement activation-related pseudoallergy (CARPA) in small animals (rodents) and large animals is fundamentally different [277], and safety trials in rodents need careful interpretation. Serum-derived exosomes from virus-infected pigs used as an intramuscular vaccine show no signs of toxicities related to CARPA [278].

There are two completed and thirteen ongoing clinical trials to evaluate the safety of exosomes. Paracrine Therapeutics Dermatology Pte. Ltd. in collaboration with National University Hospital, Singapore, completed a clinical trial on the safety and tolerability of a topical ointment prepared using mesenchymal stem cell-derived exosomes for the treatment of psoriasis (NCT05523011). The results are unreported. Other phase I and II trial evaluated the safety and efficacy of inhaled exosomes in SARS-CoV-2-associated pneumonia patients (NCT04491240) [279, 280]. The exosomes are completely safe, as no patient showed signs of toxicity. Mild inflammatory responses at the site of administration using dendritic cell-derived exosomes during tumor vaccination were reported in half of the patients during a phase I clinical trial. However, toxicity above grade 2 was not observed in any patient. These results suggest that [279, 280] even autologous exosomes may provoke CARPA.

Although numerous studies confirm the safety of exosomes in general, specific concerns with individual exosome however, need to be carefully investigated. In particular, most exosomes are derived from immortalized cell lines and may contain oncogenic molecules. Therefore, it is essential to determine whether (and to what extent) systemic repeated administrations of exosomes prompt cell transformation. Furthermore, the safety of engineered exosomes with different moieties on their surface and hybrid exosomes must be separately evaluated.

## Conclusions

Mounting evidence supports a functional role of exosomes in ovarian cancer progression, metastasis, and chemoresistance. Exosomal analysis provides key information for an early diagnosis of ovarian cancer and, permits for real-time monitoring of its progression and treatment efficiency. Their ubiquitous presence in almost all body fluids, specific distinctive features resembling their parent cells, and long-term stability during storage make them an ideal candidate for the early diagnosis of ovarian cancer. There are several different proteins, miRNAs, circular RNAs, lncRNAs, and lipids that are altered in ovarian cancer-derived exosomes that can be employed as promising biomarkers for ovarian cancer. Recent developments in microfluidics-based platforms have facilitated rapid and defined exosome isolation. Furthermore, they can merge multiple critical steps such as sample loading, processing, and detection in downstream analysis for specific RNAs and proteins on a single device. This enables the clinical translation of extracellular vesicle analysis. Single extracellular vesicle analysis is an exciting new area of research that increases the sensitivity and improves the specificity by measuring the levels of specific cancer markers on extracellular vesicles or by identifying the exclusive subpopulations of circulating extracellular vesicles.

Exosomes can be used as biomarkers in ovarian cancer and utilized for treatment. A good understanding of their role in cancer development, eliminating exosomes, or inhibiting exosome uptake by recipient cells can improve therapeutic outcomes in ovarian cancer. Exosomes from different sources can deliver cargo to ovarian cancer cells to suppress tumor growth in vivo. Therefore, they can be designed to deliver different chemo- and/or immuno-therapeutics to treat ovarian cancer. Engineering the exosome surface to prolong their half-life in circulation and to target ovarian cancer will provide added benefits. Developing hybrid exosomes is another promising area for the treatment of ovarian cancer; various hybrid exosomes have demonstrated efficacy against different tumors in vivo. A thorough safety and toxicity study of ovarian cancer-derived exosomes and their bioengineered analogues is required for their clinical translation.

**Fig. 7:**
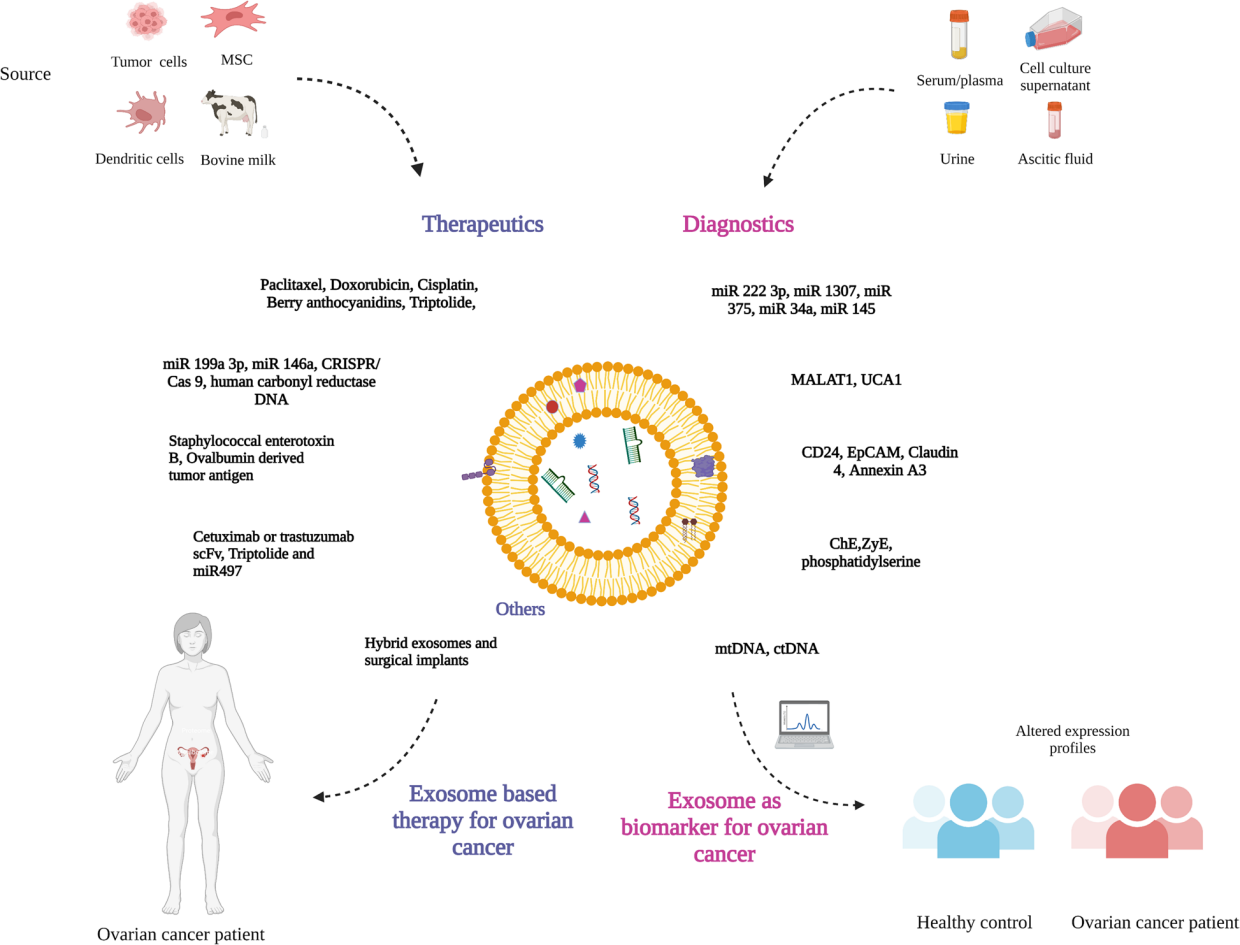
Schematic representation of therapeutic and diagnostic applications of exosomes in ovarian cancer. Image created with BioRender.com

In summary, exosome-based diagnostics and therapeutics exhibit true potential to benefit ovarian cancer patients (Fig. 7). However, exosomes face various scientific and practical obstacles to translate this technology to clinics, similar to any other novel nanomedicine-based therapy. The technology to consistently isolate large quantities of high-purity exosomes is far from standardized. There are recent improvements in exosome isolation and analysis methods; however, more precise isolation techniques are required to avoid the loss of rare exosomes and prevent the overestimation and contamination of exosomes. The exact mechanism of exosome biogenesis remains unknown. The development of more sensitive analytical methods for single extracellular vesicle analysis may be beneficial. The novel microfluidics-based isolation and analysis methods must be utilized to investigate different new ovarian cancer-related markers and validated with a high number of ovarian cancer samples to authenticate their clinical significance. The combination of a single extracellular vesicle analysis approach with cross sectional imaging can be of clinical interest. Designing of an integrated system for production of drug loaded exosomes can be of benefit with market perspective. The variety of exosomal contents that may be potentially used as biomarkers for the early detection of ovarian cancer require further validation before their clinical translation. Researchers demonstrate altered concentrations of different exosomal markers in ovarian cancer compared with those in healthy individuals; however, a valid biological range for these markers is mostly unavailable. Storage conditions can impact the physicochemical properties of exosomes. Therefore, standardized optimal storage conditions for exosomes need to be determined. Despite these hurdles, the use of exosomes may potentially overcome the existing limitations associated with the diagnosis and treatment of ovarian cancer. Finally, debate on “exosomes “ versus “ small extracellular vesicles (sEVs) “ continues to persist [281]. While ISEV recommends usage of sEVs for all particles in the size range of < 200 nm unless clearly demonstrating exosome biogenesis, articles on exosomes continues to grow and abound in the literature. Biotechnology and scientific vendors promote “exosomes”-based products as it is attractive. However, from a product development and clinical testing perspective it is important that a consensus among the scientific community studying extracellular vesicles be arrived for defining that the product is truly representive of exosomes or falls under the general category of sEVs.

## Data Availability

The authors confirm that there is no data presented in the review article and as such this section is not applicable.

## Abbreviations

APOE: Apolipoprotein E
CA 125: Carbohydrate antigen 125
CAFs: Cancer-associated fibroblasts
CARPA: Complement activation-related pseudoallergy
CCC: Clear cell carcinoma
CLDN3: Claudin 3
CSCs: Cancer stem cells
DC: Dendritic cells
DLS: Dynamic light scattering
DNMT-1: DNA methyltransferase-I
EC: Endometroid carcinoma
EGFR: Epidermal growth factor receptor
EM: Electron microscopy
EMT: Epithelial-mesenchymal transition
EpCAM: Epithelial cell surface antigen
ERBB2: Erythroblastic oncogene B
EVs: Extracellular vesicles
FASN: Fatty acid synthase
HGSC: High grade serous carcinoma
HLA: Human leukocyte antigen
HPMCs: Human peritoneal mesothelial cells
IL-6: Interleukin-6
ISEV: International Society for Extracellular Vesicles
LGSC: Low grade serous carcinoma
lncRNA: Long noncoding RNA
MC: Mucinous carcinoma
miRNA: MicroRNA
MISEV: Minimal Information for Studies on Extracellular Vesicles
MMP-9: Matrix metalloproteinase-9
MRP2: Multidrug resistance protein 2
MAGE: Melanoma antigen
MDSCs: Myeloid-derived suppressor cells
NAV: Neuron navigator
ncRNA: Non-coding RNA
NK cells: Natural killer cells
NTA: Nanoparticle tracking analysis
P-gp: P-glycoprotein
PBMC: Peripheral blood mononuclear cells
PCNA: Proliferation cell nuclear antigen
sEVs: Small extracellualr vesicles
SDF-1: Stromal derived factor-1
SEC: Size exclusion chromatography
SERS: Surface-enhanced Raman spectroscopy
siRNA: Short interfering RNA
STAT3: Signal transducer and activator of transcription 3
TAA: Tumor-associated antigens
TAM: Tumor associated macrophages
TGF-β1: Transforming growth factor β-1
TME: Tumor microenvironment
TMT: Tandem mass tagging
TRAIL: TNF-related apoptosis-inducing ligand
TUBB3: Tubulin B-3 chain
